# The Global Prevalence of Amblyopia in Children: A Systematic Review and Meta-Analysis

**DOI:** 10.3389/fped.2022.819998

**Published:** 2022-05-04

**Authors:** Budan Hu, Zongshun Liu, Jiao Zhao, Li Zeng, Gengsheng Hao, Dan Shui, Ke Mao

**Affiliations:** Department of Ophthalmology, The People’s Hospital of Leshan, Leshan, China

**Keywords:** amblyopia, children, prevalence, worldwide, meta-analysis

## Abstract

Epidemiological data about the prevalence of amblyopia around the world vary widely among regions and periods. This meta-analysis aimed to determine the global prevalence of amblyopia in children. PubMed, Embase, and the Cochrane Library were searched for prevalence studies published up to 5 November 2021. The outcome was the prevalence of amblyopia, analyzed as pooled estimates with 95% confidence intervals (CI). A total of 97 studies were included, including 4,645,274 children and 7,706 patients with amblyopia. The overall worldwide pooled prevalence of amblyopia was 1.36% (95%CI: 1.27–1.46%). The prevalence of amblyopia was higher in males (1.40%, 95%CI: 1.10–1.70%) than in females (1.24%, 95%CI: 0.94–1.54%) (*OR* = 0.885, 95%CI: 0.795–0.985, *P* = 0.025). The results of the meta-regression analysis showed that there were no significant associations between the prevalence of amblyopia and geographical area, publication year, age, sample size, and whether it was carried out in a developed or developing country (all *P* > 0.05). Begg’s test (*P* = 0.065) and Egger’s test (*P* < 0.001) showed that there was a significant publication bias in the prevalence of amblyopia. In conclusion, amblyopia is a significant vision problem worldwide, and public health strategies of early screening, treatment, and management are important.

## Introduction

Amblyopia is a common vision disorder among children and is defined as decreased vision due to abnormal development of the visual cortex in infancy or childhood. Amblyopia is a reduction in best-corrected visual acuity (BCVA) (2-line difference between the two eyes) secondary to neurological deficits in visual output caused by abnormal brain stimulation during critical periods of visual development (1–3). It is usually unilateral and is the most common cause of vision loss and mononuclear blindness in children (1–3). It can be caused by any condition that creates a disparity in vision between the two eyes (1), and 90% of the cases are reportedly caused by strabismus and/or anisometropia (1–3). The treatment is characterized by the occlusion and penalization of the better-seeing eye while forcing the use of the amblyopic eye (3). The initial treatment includes refractive correction of visual impairment in the affected eye(s) with eyeglasses, the correction of any strabismus with glasses or surgery if severe, and the removal of any obstacle to vision, such as cataract or local hemangioma (3). Possible complications include the permanent loss of vision in the affected eye (1, 2), estimated at 1.2% lifetime risk (4). If detected early, most patients will have normal vision restored (2).

The reported incidence is 1–5% worldwide and 2–4% in North America (2, 3), but the reported prevalence varies widely among studies, from 0.05 to 7.54% (5–21). Those studies are from various countries and different periods and included children of different age groups. Therefore, this data must be considered to be highly fragmented. A meta-analysis of 73 studies, published in 2018, showed that the pooled prevalence of amblyopia was 1.75%, varying from 0.51% in Africa to 3.67% in Europe (22). A meta-analysis of 60 studies, published in 2019, reported a pooled prevalence of 1.44% among children and young adults, with 0.72% in Africa, 1.09% in Asia, 2.41% in America, and 2.90% in Europe (23). Since the publication of these meta-analyses, novel studies have been published that could help improve the global estimates.

Therefore, the purpose of this meta-analysis was to determine the global prevalence of amblyopia in children. Improvements in screening methods and policies might lead to changes in the prevalence of amblyopia, which could be important for public health and decision-makers. More accurate estimates might be important for policymakers in public health.

## Methods

### Literature Search

This meta-analysis was performed in accordance with the Preferred Reporting Items for Systematic Reviews and Meta-Analyses (PRISMA) reporting guidelines (24). Since no original clinical raw data was collected or used, ethical approval was not requested for this meta-analysis.

Three recognized electronic databases, PubMed, Embase, and the Cochrane Library, were searched for studies published from inception up to 5 November 2021, using the MeSH terms of “child,” “amblyopia,” “prevalence,” and “epidemiology” combined with relevant key words. The eligibility criteria were (1) study type: prevalence study (because this study aimed to summarize the global prevalence), (2) population: since amblyopia occurs in childhood, only studies reporting data in juveniles/children were included (because the definition of children or juvenile varies among countries, a study could be included as long as the authors considered their study population to be underage), (3) outcome: prevalence of amblyopia, and (4) full text published in English. Special groups, such as hospitalized patients or patients with certain ocular or systemic diseases, were excluded. If prevalence data or overlapping groups of participants were reported in multiple papers, the original paper was selected for inclusion.

### Data Extraction and Quality Assessment

Potentially relevant publications were screened and evaluated by two reviewers (Budan Hu and Li Zeng) in a double-blind manner, with a third reviewer (Gengsheng Hao, Dan Shui, or Ke Mao) resolving any disagreement. A structured data collection form was developed. Two researchers (Zongshun Liu and Jiao Zhao) independently extracted the data, including authors, year of publication, country, study design, sample size, age, percentage of males, the definition of amblyopia, number of cases, number of subjects, and prevalence of amblyopia. “Mixed country” referred to studies that included countries from different continents.

The cross-sectional studies were evaluated using the Healthcare Research and Quality (AHRQ) tool (25). The cohort studies were evaluated according to the Newcastle-Ottawa scale (NOS) (26).

### Statistical Analysis

All analyses were performed using STATA MP 14.0 (StataCorp, College Station, TX, United States). Prevalence with the 95% confidence interval (CI) was combined for statistical analysis. Prevalence estimates were converted using the Freeman-Tukey transformation and back-transformed after quantitative data synthesis (27). Statistical heterogeneity among studies was calculated using Cochran’s Q-test and the I^2^ index. An *I*^2^ > 50% and Q-test *P* < 0.10 indicated high heterogeneity, and the random-effects model was used; otherwise, the fixed-effects model was applied. In the case of the random-effects model, the tau square was calculated as instructed in the Cochrane Handbook (28). *P*-values = 0.05 were considered statistically significant. Potential publication bias (resulting from the publication or non-publication of relevant trials) was assessed by funnel plots, Egger’s test, and Begg’s test (28–30). Univariable meta-regression models were used to investigate the effect of age, sample size, publication year, developed or developing region, and geographical location as factors affecting the prevalence of amblyopia. Subgroups were compared using odds ratios (OR) and 95%CI.

## Results

### Selection of the Studies

**FIGURE 1:**
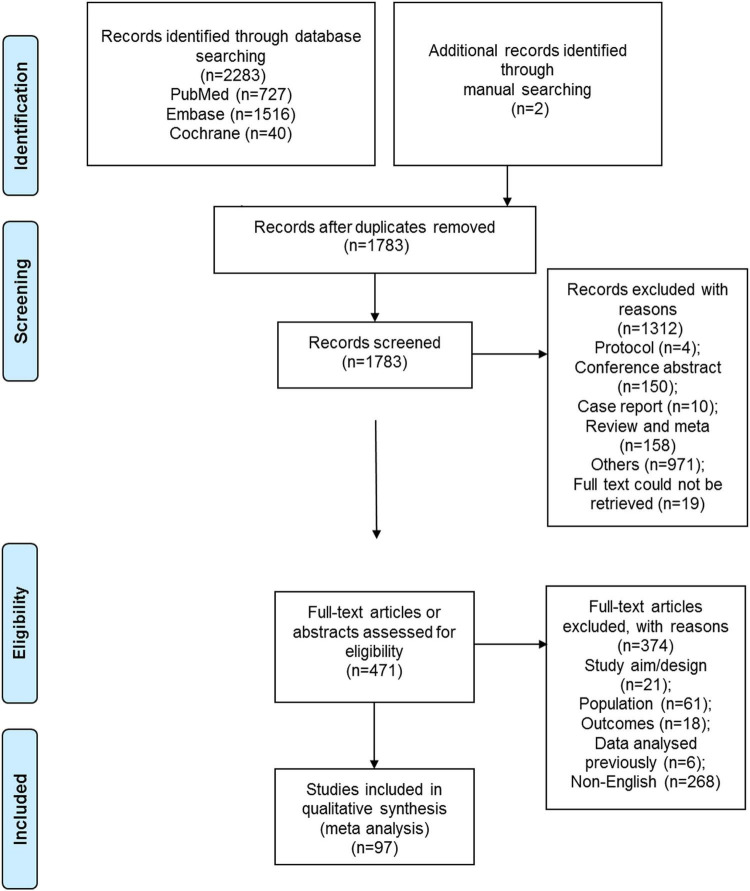
Flowchart of the search process.

Figure 1 shows the selection process. A total of 2,283 records were retrieved, and 1,783 were left after removing the duplicates. Supplementary Figure 1 presents the search strings for PubMed. From these, 1,312 were excluded after screening the titles and abstracts, and 471 full-text papers were assessed for eligibility. From them, 21 were excluded because of study aim or design, 61 because of the study population, 18 because of the outcomes, 6 because of previously analyzed data, and 268 for non-English full-text papers. Finally, 97 studies were included in the present meta-analysis (Supplementary Table 2). A total of 4,645,274 children were included, including 7,706 patients with amblyopia. The study quality assessments are presented in Supplementary Tables 3a,b.

### Pooled Prevalence of Amblyopia

**FIGURE 2:**
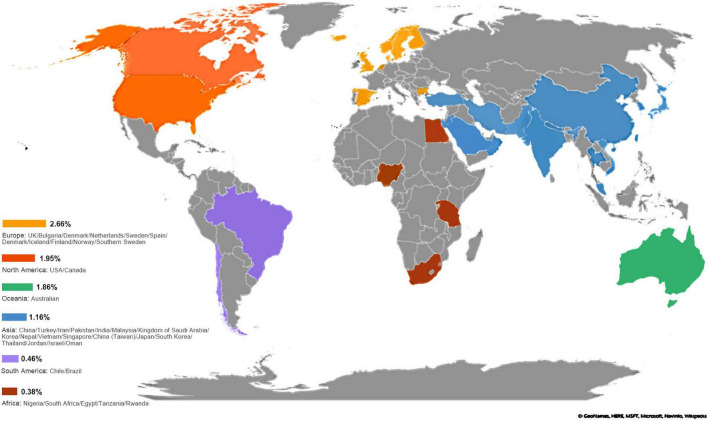
Pooled prevalence of amblyopia based on countries. The reported prevalence values were pooled for each country. A darker shade of blue indicates higher prevalence. No data were available for the countries in gray.

**FIGURE 3:**
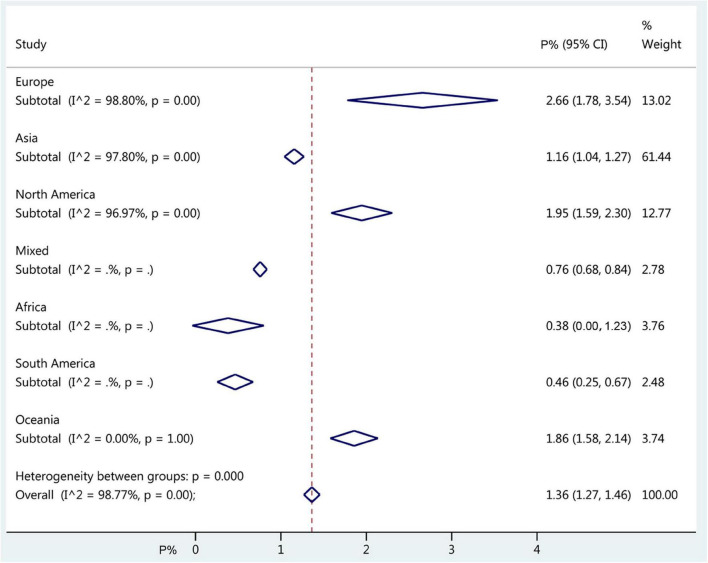
Forest plot of pooled continent prevalence of amblyopia. The diamonds represent the prevalence estimates with 95% confidence intervals (CIs). The dashed red line represents the global estimate. The *x*-axis represents the prevalence of amblyopia, in%. “Mixed country” referred to studies that included countries from different continents.

The overall worldwide pooled prevalence of amblyopia was 1.36% (95%CI: 1.27–1.46%; *I*^2^ = 98.8%, P_heterogeneity_ < 0.01). When considering each continent, the pooled prevalence of amblyopia was 2.66% (95%CI: 1.78–3.54%; *I*^2^ = 98.8%, P_heterogeneity_ < 0.01) in Europe, 1.95% (95%CI: 1.59–2.30%; *I*^2^ = 97.0%, P_heterogeneity_ < 0.01) in North America, 1.86% (95%CI: 1.58–2.14%; *I*^2^ = 0.0%, P_heterogeneity_ > 0.99) in Oceania, 1.16% (95%CI: 1.04–1.27%; *I*^2^ = 97.8%, P_heterogeneity_ < 0.01) in Asia, 0.46% (95%CI: 0.25–0.67%) in South America, 0.38% (95%CI: 0.00–1.23%) in Africa, and 0.76% (95%CI: 0.68–0.84%) in mixed countries (Figures 2, 3, Supplementary Figure 1, and Table 1).

**TABLE 1:** Subgroup results.

	No. of studies	No. of cases	No. of subjects	Pooled prevalence (%)	95%CI	P	I^2^	P_heterogeneity_
Overall	97	7,745	4,645,274	1.36	1.27–1.46	<0.01	98.77%	<0.01
**Continent**								
Europe	16	1,265	3,858,073	2.66	1.78–3.54	<0.01	98.80%	<0.01
Asia	57	3,908	555,919	1.16	1.04–1.27	<0.01	97.80%	<0.01
North America	13	2,009	168,000	1.95	1.59–2.3	<0.01	96.97%	<0.01
Africa	3	22	5,006	0.38	0.00–1.23	0.014	NA	NA
South America	2	29	4,028	0.46	0.25–0.67	<0.01	NA	NA
Oceania	4	168	9,043	1.86	1.58–2.14	<0.01	0.00%	1.00
Mixed	2	344	45,205	0.76	0.68–0.84	<0.01	NA	NA
**Gender**								
Male	21	1,381	181,168	1.4	1.1–1.7	<0.01	94.25%	<0.01
Female	21	1,381	181,168	1.24	0.94–1.54	<0.01	95.58%	<0.01
**Publication year**								
2011 or after	50	4,914	468,878	1.45	1.27–1.63	<0.01	98.32%	<0.01
2010 or before	47	2,831	4,176,396	1.29	1.17–1.4	<0.01	98.38%	<0.01
**Country development**								
Developed	44	4,157	4,320,999	1.52	1.39–1.65	<0.01	98.97%	<0.01
Developing	53	3,588	324,275	1.35	1.16–1.55	<0.01	97.82%	<0.01
**Uni/bilateral**								
Unilateral	25	1,450	185,443	1.08	0.92–1.24	<0.01	91.61%	<0.01
Bilateral	25	471	185,443	0.31	0.24–0.37	<0.01	82.75%	<0.01
**Cause**								
Isoametropic	8	169	247,873	0.09	0.05–0.13	<0.01	93.90%	<0.01
Anisometropic	18	708	290,245	0.47	0.38–0.55	<0.01	96.99%	<0.01
Strabismic	18	373	267,723	0.17	0.13–0.21	<0.01	94.83%	<0.01

*“Mixed country” referred to studies that included countries from different continents.*

### Subgroup Analyses

**FIGURE 4:**
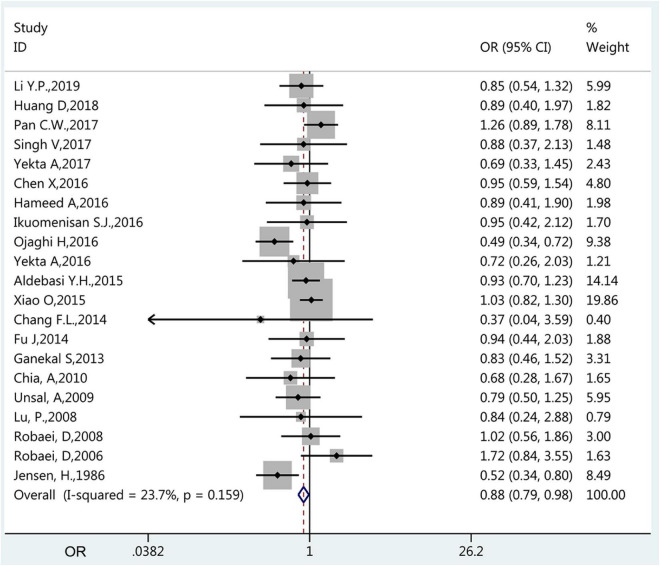
Forest plot of prevalence of amblyopia of female vs. male. The small diamonds are the odds ratios (ORs), the line represents the 95% confidence intervals (CIs), and the gray boxes represent the proportional sample size. The *x*-axis represents the ORs. This analysis only included the studies (*n* = 20) that compared males vs. females.

The prevalence of amblyopia was higher in males (1.40%, 95%CI: 1.10–1.70%; *I*^2^ = 94.3%, P_heterogeneity_ < 0.01) than in females (1.24%, 95%CI: 0.94–1.54%; *I*^2^ = 95.6%, P_heterogeneity_ < 0.01) (*OR* = 0.885, 95%CI: 0.795–0.985, *P* = 0.025) (Figure 4 and Table 1). The results of the meta-regression analysis showed that there were no significant associations between the prevalence of amblyopia and geographical area, publication year, age, sample size, and whether it was carried out in a developed or developing country (Table 2).

**TABLE 2:** Result of univariable meta-regression analysis.

Variable	Coefficient	95%CI	*P*
**Continent**			
Africa	−0.0035939	−0.0305802 to 0.0233925	0.792
Asia	0.0063657	−0.0148711 to 0.0276024	0.553
Europe	0.0180953	−0.0041382 to 0.0403289	0.109
North America	0.014575	−0.0079233 to 0.0370733	0.201
Oceania	0.0103513	−0.0153681 to 0.0360706	0.426
South America	−0.0019128	−0.0314849 to 0.0276593	0.898
Publication year	−8.84E-06	−0.000362 to 0.0003443	0.96
Age	−0.0008564	−0.0018221 to 0.0001094	0.082
Sample size	−5.02E-09	−1.31e-08 to 3.03e-09	0.219
Developed or developing	0.0046731	−0.0016898 to 0.0110361	0.148

### Assessment of Publication Bias

Begg’s test (*P* = 0.065) and Egger’s test (*P* < 0.001) showed that there was a significant publication bias in the prevalence of amblyopia.

## Discussion

Epidemiological data about the prevalence of amblyopia around the world vary widely among regions and periods. Therefore, the present meta-analysis aimed to determine the global prevalence of amblyopia in children. The results indicate that the prevalence of amblyopia varies between boys and girls, but not according to the geographical area, publication year, age, sample size, and economic status. Nevertheless, it still is a significant vision problem worldwide, and public health strategies of early screening, treatment, and management are important.

In the present study, the worldwide pooled prevalence of amblyopia was 1.36%. It is supported by previous meta-analyses as it is within the range of the reported pooled prevalence rates, with 1.44% for Fu et al. (23) and 1.75% for Hashemi et al. (22). Indeed, Simons et al. (31) reported in 2005 a worldwide prevalence of 1.6–3.6%.

Xiao et al. (32) reported a prevalence of 0.625%, while other studies in China reported a prevalence of around 1.19% (11, 33, 34). High prevalence in Europe has been reported (35, 36) and the United States of America (37–39). Hashemi et al. (22) reported that the pooled prevalence of amblyopia varied from 0.51% in Africa to 3.67% in Europe, while Fu et al. (23) reported that the pooled prevalence of amblyopia was 0.72% in Africa, 1.09% in Asia, 2.41% in America, and 2.90% in Europe. The present meta-analysis observed similar trends, with 2.66% in Europe, 1.95% in North America, 1.86% in Oceania, 1.16% in Asia, 0.46% in South America, and 0.38% in Africa. The meta-regression analysis showed that those differences were not statistically significant. Nevertheless, those apparent differences might be explained by the socio-economic status of the continents, leading to different access to screening. It is in contrast with the study by Hashemi et al. (22), whose meta-regression analysis revealed differences among continents, and by other studies as well (32, 38, 40–42), but Simons et al. (31) rejected the presence of ethnic differences in the prevalence of amblyopia, supporting the present study.

The present meta-analysis revealed a significant difference in the prevalence of amblyopia between boys and girls, with a higher prevalence in boys. It is in contradiction with Fu et al. (23), who reported a higher prevalence in girls. A study from Nigeria reported that all cases were males (43). Nevertheless, sex is not recognized as a risk factor for amblyopia (1–3), and those differences warrant further study.

In the present study, there were no significant differences among the causes of amblyopia. Previous studies reported that anisometropia was the most common cause of amblyopia (22, 31, 36), which is fortunate because it is the most easily treatable form of amblyopia (31), involving mostly optical correction without occlusion or penalization. Nevertheless, a source of bias that could explain the lack of differences among the causes of amblyopia in the present study could be that the causes vary according to ethnicity. Indeed, strabismus might be more common in non-Hispanic Caucasians than in Asians (44, 45), while anisometropic amblyopia could be more prevalent in the Middle East (46, 47).

Previous studies reported differences in the prevalence of amblyopia among periods, probably due to more or less attention given to the disease, public awareness, and screening programs (23, 48, 49). Such a difference was not observed in the present meta-analysis.

In the present study, the publication bias was high. It is supported by the recent previous meta-analyses on the prevalence of amblyopia worldwide (22, 23).

Of course, the results of the present meta-analysis must be considered alongside its limitations. Indeed, despite the high numbers of included studies and children, nearly all analyses suffered from significantly high heterogeneity. This high heterogeneity is probably rooted in the different countries, periods, diagnostic tools, screening policies, and economic status. Nevertheless, the subgroup results must be taken with caution since some subgroups contained only a small number of studies/participants. Second, the definition of juvenile/child varies among countries, and the studies were included if their authors considered their study population to be a child. Therefore, some patients were included but would be considered young adults by some authors. It had to be done because, without access to the raw data, it would be impossible to exclude older participants specifically. Third, not all studies reported differences between boys and girls, which could bias the results.

In conclusion, the present meta-analysis indicates that the prevalence of amblyopia varies between boys and girls, but not according to the geographical area, publication year, age, sample size, and economic status. Nevertheless, it still is a significant vision problem worldwide, and public health strategies of early screening, treatment, and management are important.

## Data Availability Statement

The original contributions presented in the study are included in the article/Supplementary Material, further inquiries can be directed to the corresponding authors.

## Author Contributions

BH and LZ carried out the studies, participated in collecting data, and drafted the manuscript. ZL and JZ performed the statistical analysis and participated in its design. GH, DS, and KM helped to draft the manuscript. All authors read and approved the final manuscript.

## Conflict of Interest

The authors declare that the research was conducted in the absence of any commercial or financial relationships that could be construed as a potential conflict of interest.

## Publisher’s Note

All claims expressed in this article are solely those of the authors and do not necessarily represent those of their affiliated organizations, or those of the publisher, the editors and the reviewers. Any product that may be evaluated in this article, or claim that may be made by its manufacturer, is not guaranteed or endorsed by the publisher.
