# Supplementary material for: The Global Prevalence of Amblyopia in Children: A Systematic Review and Meta-Analysis
Source: Front Pediatr. 2022 May 4;10:819998. doi: 10.3389/fped.2022.819998 (PMC9114436; doi:10.3389/fped.2022.819998)

The global prevalence of amblyopia in children: a systematic review and meta-analysis

Running title: Worldwide prevalence of amblyopia in minors

Budan Hu†, Zongshun Liu†, Jiao Zhao, Li Zeng, Gengsheng Hao, Dan Shui, Ke Mao

Department of Ophthalmology, The People's Hospital of Leshan, Leshan, Sichuan, China

†These authors contributed equally to this work.

***Co-corresponding authors:**

Budan Hu

Department of Ophthalmology, The People's Hospital of Leshan, Leshan, Sichuan, China

Tel: +86-13881343051

E-mail: 543497114@qq.com

Zongshun Liu

Department of Ophthalmology, The People's Hospital of Leshan, Leshan, Sichuan, China

E-mail: liuzongshun0507@126.com

SUPPLEMENTARY MATERIALS

Supplemental Table S1. Search process for PubMed

Search number, Query, Sort By, Filters, Search Details, Results, Time
28,"(((child[MeSH Terms]) OR (children[Title/Abstract] OR pediatric[Title/Abstract])) AND ((Amblyopia[MeSH Terms]) OR (Amblyopias[Title/Abstract] OR Lazy Eye[Title/Abstract] OR Eye, Lazy[Title/Abstract] OR Eyes, Lazy[Title/Abstract] OR Lazy Eyes[Title/Abstract] OR Anisometropic Amblyopia[Title/Abstract] OR Amblyopia, Anisometropic[Title/Abstract] OR Amblyopias, Anisometropic[Title/Abstract] OR Anisometropic Amblyopias[Title/Abstract] OR Amblyopia, Developmental[Title/Abstract] OR Amblyopias, Developmental[Title/Abstract] OR Developmental Amblyopia[Title/Abstract] OR Developmental Amblyopias[Title/Abstract] OR Amblyopia, Suppression[Title/Abstract] OR Amblyopias, Suppression[Title/Abstract] OR Suppression Amblyopia[Title/Abstract] OR Suppression Amblyopias[Title/Abstract] OR Stimulus Deprivation-Induced Amblyopia[Title/Abstract] OR Stimulus Deprivation Induced Amblyopia[Title/Abstract] OR Amblyopia, Stimulus Deprivation-Induced[Title/Abstract] OR Amblyopia, Stimulus Deprivation Induced[Title/Abstract] OR Amblyopias, Stimulus Deprivation-Induced[Title/Abstract] OR Deprivation-Induced Amblyopia, Stimulus[Title/Abstract] OR Deprivation-Induced Amblyopias, Stimulus[Title/Abstract] OR Stimulus Deprivation-Induced Amblyopias[Title/Abstract]))) AND (((Prevalence[MeSH Terms]) OR (Epidemiology[MeSH Terms]) OR ""epidemiology"" [Subheading]) OR (Prevalences[Title/Abstract] OR prevalence study[Title/Abstract] OR epidemics[Title/Abstract] OR frequency[Title/Abstract] OR surveillance[Title/Abstract] OR morbidity[Title/Abstract] OR occurrence[Title/Abstract] OR outbreaks[Title/Abstract] OR prevalence[Title/Abstract] OR endemics[Title/Abstract] OR incidence[Title/Abstract]))",,,"((""child""[MeSH Terms] OR (""children""[Title/Abstract] OR ""pediatric""[Title/Abstract])) AND (""Amblyopia""[MeSH Terms] OR (((((((((((((((((((((((((""Amblyopias""[Title/Abstract] OR ""lazy eye""[Title/Abstract]) OR ""eye lazy""[Title/Abstract]) OR (((""Eye""[MeSH Terms] OR ""Eye""[All Fields]) OR ""Eyes""[All Fields]) AND ""Lazy""[Title/Abstract])) OR ""lazy eyes""[Title/Abstract]) OR ""anisometropic amblyopia""[Title/Abstract]) OR ""amblyopia anisometropic""[Title/Abstract]) OR ""amblyopias anisometropic""[Title/Abstract]) OR ""anisometropic amblyopias""[Title/Abstract]) OR (((""Amblyopia""[MeSH Terms] OR ""Amblyopia""[All Fields]) OR ""Amblyopias""[All Fields]) AND ""Developmental""[Title/Abstract])) OR (((""Amblyopia""[MeSH Terms] OR ""Amblyopia""[All Fields]) OR ""Amblyopias""[All Fields]) AND ""Developmental""[Title/Abstract])) OR ((""Developmental""[All Fields] OR ""developmentally""[All Fields]) AND ""Amblyopia""[Title/Abstract])) OR ((""Developmental""[All Fields] OR ""developmentally""[All Fields]) AND ""Amblyopias""[Title/Abstract])) OR ""amblyopia suppression""[Title/Abstract]) OR (((""Amblyopia""[MeSH Terms] OR ""Amblyopia""[All Fields]) OR ""Amblyopias""[All Fields]) AND ""Suppression""[Title/Abstract])) OR ""suppression amblyopia""[Title/Abstract]) OR (((((((((((""suppress""[All Fields] OR ""suppressed""[All Fields]) OR ""suppresser""[All Fields]) OR ""suppresses""[All Fields]) OR ""suppressibility""[All Fields]) OR ""suppressible""[All Fields]) OR ""suppressing""[All Fields])

OR ""Suppression""[All Fields]) OR ""suppressions""[All Fields]) OR ""suppressive""[All Fields]) OR ""suppressives""[All Fields]) AND ""Amblyopias""[Title/Abstract])) OR (((""Stimulus""[All Fields] OR ""stimulus's""[All Fields]) AND ""Deprivation-Induced""[All Fields]) AND ""Amblyopia""[Title/Abstract])) OR (((""Stimulus""[All Fields] OR ""stimulus's""[All Fields]) AND ((((((""deprival""[All Fields] OR ""Deprivation""[All Fields]) OR ""deprivations""[All Fields]) OR ""deprive""[All Fields]) OR ""deprived""[All Fields]) OR ""deprives""[All Fields]) OR ""depriving""[All Fields])) AND ""induced amblyopia""[Title/Abstract])) OR ((((""Amblyopia""[MeSH Terms] OR ""Amblyopia""[All Fields]) OR ""Amblyopias""[All Fields]) AND (""Stimulus""[All Fields] OR ""stimulus's""[All Fields])) AND ""Deprivation-Induced""[Title/Abstract])) OR ((((""Amblyopia""[MeSH Terms] OR ""Amblyopia""[All Fields]) OR ""Amblyopias""[All Fields]) AND (""Stimulus""[All Fields] OR ""stimulus's""[All Fields])) AND ""Deprivation-Induced""[Title/Abstract])) OR ((((""Amblyopia""[MeSH Terms] OR ""Amblyopia""[All Fields]) OR ""Amblyopias""[All Fields]) AND (""Stimulus""[All Fields] OR ""stimulus's""[All Fields])) AND ""Deprivation-Induced""[Title/Abstract])) OR (""Deprivation-Induced""[All Fields] AND ""amblyopia stimulus""[Title/Abstract])) OR ((""Deprivation-Induced""[All Fields] AND ((""Amblyopia""[MeSH Terms] OR ""Amblyopia""[All Fields]) OR ""Amblyopias""[All Fields])) AND ""Stimulus""[Title/Abstract])) OR (((""Stimulus""[All Fields] OR ""stimulus's""[All Fields]) AND ""Deprivation-Induced""[All Fields]) AND ""Amblyopias""[Title/Abstract])))) AND (((""prevalence""[MeSH Terms] OR ""epidemiology""[MeSH Terms]) OR ""epidemiology""[MeSH Subheading]) OR ((((((((((""Prevalences""[Title/Abstract] OR ""prevalence study""[Title/Abstract]) OR ""epidemics""[Title/Abstract]) OR ""frequency""[Title/Abstract]) OR ""surveillance""[Title/Abstract]) OR ""morbidity""[Title/Abstract]) OR ""occurrence""[Title/Abstract]) OR ""outbreaks""[Title/Abstract]) OR ""prevalence""[Title/Abstract]) OR ""endemics""[Title/Abstract]) OR ""incidence""[Title/Abstract]))",727,21:24:28
27,"((Prevalence[MeSH Terms]) OR (Epidemiology[MeSH Terms]) OR ""epidemiology"" [Subheading]) OR (Prevalences[Title/Abstract] OR prevalence study[Title/Abstract] OR epidemics[Title/Abstract] OR frequency[Title/Abstract] OR surveillance[Title/Abstract] OR morbidity[Title/Abstract] OR occurrence[Title/Abstract] OR outbreaks[Title/Abstract] OR prevalence[Title/Abstract] OR endemics[Title/Abstract] OR incidence[Title/Abstract])",,,"""prevalence""[MeSH Terms] OR ""epidemiology""[MeSH Terms] OR ""epidemiology""[MeSH Subheading] OR ""Prevalences""[Title/Abstract] OR ""prevalence study""[Title/Abstract] OR ""epidemics""[Title/Abstract] OR ""frequency""[Title/Abstract] OR ""surveillance""[Title/Abstract] OR ""morbidity""[Title/Abstract] OR ""occurrence""[Title/Abstract] OR ""outbreaks""[Title/Abstract] OR ""prevalence""[Title/Abstract] OR ""endemics""[Title/Abstract] OR ""incidence""[Title/Abstract]","4,063,488",21:24:15
25,"(Amblyopia[MeSH Terms]) OR (Amblyopias[Title/Abstract] OR Lazy Eye[Title/Abstract] OR Eye, Lazy[Title/Abstract] OR Eyes, Lazy[Title/Abstract] OR Lazy Eyes[Title/Abstract] OR Anisometropic Amblyopia[Title/Abstract] OR Amblyopia, Anisometropic[Title/Abstract] OR Amblyopias, Anisometropic[Title/Abstract] OR Anisometropic Amblyopias[Title/Abstract] OR Amblyopia, Developmental[Title/Abstract] OR Amblyopias, Developmental[Title/Abstract] OR Developmental Amblyopia[Title/Abstract] OR

Developmental Amblyopias[Title/Abstract] OR Amblyopia, Suppression[Title/Abstract] OR Amblyopias, Suppression[Title/Abstract] OR Suppression Amblyopia[Title/Abstract] OR Suppression Amblyopias[Title/Abstract] OR Stimulus Deprivation-Induced Amblyopia[Title/Abstract] OR Stimulus Deprivation Induced Amblyopia[Title/Abstract] OR Amblyopia, Stimulus Deprivation-Induced[Title/Abstract] OR Amblyopia, Stimulus Deprivation Induced[Title/Abstract] OR Amblyopias, Stimulus Deprivation-Induced[Title/Abstract] OR Deprivation-Induced Amblyopia, Stimulus[Title/Abstract] OR Deprivation-Induced Amblyopias, Stimulus[Title/Abstract] OR Stimulus Deprivation-Induced Amblyopias[Title/Abstract])",,,"""Amblyopia""[MeSH Terms] OR ((((((((((((((((((((((""Amblyopias""[Title/Abstract] OR ""lazy eye""[Title/Abstract]) OR ""eye lazy""[Title/Abstract]) OR (((""Eye""[MeSH Terms] OR ""Eye""[All Fields]) OR ""Eyes""[All Fields]) AND ""Lazy""[Title/Abstract])) OR ""lazy eyes""[Title/Abstract]) OR ""anisometropic amblyopia""[Title/Abstract]) OR ""amblyopia anisometropic""[Title/Abstract]) OR ""amblyopias anisometropic""[Title/Abstract]) OR ""anisometropic amblyopias""[Title/Abstract]) OR (((""Amblyopia""[MeSH Terms] OR ""Amblyopia""[All Fields]) OR ""Amblyopias""[All Fields]) AND ""Developmental""[Title/Abstract])) OR (((""Amblyopia""[MeSH Terms] OR ""Amblyopia""[All Fields]) OR ""Amblyopias""[All Fields]) AND ""Developmental""[Title/Abstract])) OR ((""Developmental""[All Fields] OR ""developmentally""[All Fields]) AND ""Amblyopia""[Title/Abstract])) OR ((""Developmental""[All Fields] OR ""developmentally""[All Fields]) AND ""Amblyopias""[Title/Abstract])) OR ""amblyopia suppression""[Title/Abstract]) OR (((""Amblyopia""[MeSH Terms] OR ""Amblyopia""[All Fields]) OR ""Amblyopias""[All Fields]) AND ""Suppression""[Title/Abstract])) OR ""suppression amblyopia""[Title/Abstract]) OR ((((((((((""suppress""[All Fields] OR ""suppressed""[All Fields]) OR ""suppresser""[All Fields]) OR ""suppresses""[All Fields]) OR ""suppressibility""[All Fields]) OR ""suppressible""[All Fields]) OR ""suppressing""[All Fields]) OR ""Suppression""[All Fields]) OR ""suppressions""[All Fields]) OR ""suppressive""[All Fields]) OR ""suppressives""[All Fields]) AND ""Amblyopias""[Title/Abstract])) OR (((""Stimulus""[All Fields] OR ""stimulus's""[All Fields]) AND ""Deprivation-Induced""[All Fields]) AND ""Amblyopia""[Title/Abstract])) OR (((""Stimulus""[All Fields] OR ""stimulus's""[All Fields]) AND ((((((""deprival""[All Fields] OR ""Deprivation""[All Fields]) OR ""deprivations""[All Fields]) OR ""deprive""[All Fields]) OR ""deprived""[All Fields]) OR ""deprives""[All Fields]) OR ""depriving""[All Fields])) AND ""induced amblyopia""[Title/Abstract])) OR ((((""Amblyopia""[MeSH Terms] OR ""Amblyopia""[All Fields]) OR ""Amblyopias""[All Fields]) AND (""Stimulus""[All Fields] OR ""stimulus's""[All Fields])) AND ""Deprivation-Induced""[Title/Abstract])) OR ((((""Amblyopia""[MeSH Terms] OR ""Amblyopia""[All Fields]) OR ""Amblyopias""[All Fields]) AND (""Stimulus""[All Fields] OR ""stimulus's""[All Fields])) AND ""Deprivation-Induced""[Title/Abstract])) OR ((((""Amblyopia""[MeSH Terms] OR ""Amblyopia""[All Fields]) OR ""Amblyopias""[All Fields]) AND (""Stimulus""[All Fields] OR ""stimulus's""[All Fields])) AND ""Deprivation-Induced""[Title/Abstract])) OR (""Deprivation-Induced""[All Fields] AND ""amblyopia stimulus""[Title/Abstract])) OR ((""Deprivation-Induced""[All Fields] AND ((""Amblyopia""[MeSH Terms] OR ""Amblyopia""[All Fields]) OR ""Amblyopias""[All Fields])) AND ""Stimulus""[Title/Abstract])) OR (((""Stimulus""[All Fields] OR ""stimulus's""[All Fields]) AND ""Deprivation-Induced""[All Fields]) AND ""Amblyopias""[Title/Abstract]))","6,946",21:21:25

20,(child[MeSH Terms]) OR (children[Title/Abstract] OR pediatric[Title/Abstract]),,,"""child""[MeSH Terms] OR ""children""[Title/Abstract] OR ""pediatric""[Title/Abstract]","2,223,050",21:17:40
17,"((((child[MeSH Terms]) OR (children)) OR (pediatric)) AND ((Amblyopia[MeSH Terms]) OR ((Amblyopias) OR (Lazy Eye) OR (Eye, Lazy) OR (Eyes, Lazy) OR (Lazy Eyes) OR (Anisometropic Amblyopia) OR (Amblyopia, Anisometropic) OR (Amblyopias, Anisometropic) OR (Anisometropic Amblyopias) OR (Amblyopia, Developmental) OR (Amblyopias, Developmental) OR (Developmental Amblyopia) OR (Developmental Amblyopias) OR (Amblyopia, Suppression) OR (Amblyopias, Suppression) OR (Suppression Amblyopia) OR (Suppression Amblyopias) OR (Stimulus Deprivation-Induced Amblyopia) OR (Stimulus Deprivation Induced Amblyopia) OR (Amblyopia, Stimulus Deprivation-Induced) OR (Amblyopia, Stimulus Deprivation Induced) OR (Amblyopias, Stimulus Deprivation-Induced) OR (Deprivation-Induced Amblyopia, Stimulus) OR (Deprivation-Induced Amblyopias, Stimulus) OR (Stimulus Deprivation-Induced Amblyopias)))) AND (((Prevalence[MeSH Terms]) OR (Prevalences) OR (prevalence study)) OR (""Epidemiology""[Mesh] OR ""epidemiology"" [Subheading] OR epidemics OR frequency OR surveillance OR morbidity OR occurrence OR outbreaks OR prevalence OR endemics OR incidence))",,,"(((""child""[MeSH Terms] OR ((((((""child""[MeSH Terms] OR ""child""[All Fields]) OR ""children""[All Fields]) OR ""child's""[All Fields]) OR ""children's""[All Fields]) OR ""childrens""[All Fields]) OR ""childs""[All Fields])) OR ((((""paediatrics""[All Fields] OR ""pediatrics""[MeSH Terms]) OR ""pediatrics""[All Fields]) OR ""paediatric""[All Fields]) OR ""pediatric""[All Fields])) AND (""Amblyopia""[MeSH Terms] OR (((((((((((((((((((((((((((""Amblyopia""[MeSH Terms] OR ""Amblyopia""[All Fields]) OR ""Amblyopias""[All Fields]) OR (((""Amblyopia""[MeSH Terms] OR ""Amblyopia""[All Fields]) OR (""Lazy""[All Fields] AND ""Eye""[All Fields])) OR ""lazy eye""[All Fields])) OR (((""Amblyopia""[MeSH Terms] OR ""Amblyopia""[All Fields]) OR (""Eye""[All Fields] AND ""Lazy""[All Fields])) OR ""eye lazy""[All Fields])) OR (((""Amblyopia""[MeSH Terms] OR ""Amblyopia""[All Fields]) OR (""Eyes""[All Fields] AND ""Lazy""[All Fields])))) OR (((""Amblyopia""[MeSH Terms] OR ""Amblyopia""[All Fields]) OR (""Lazy""[All Fields] AND ""Eyes""[All Fields])) OR ""lazy eyes""[All Fields])) OR (((""Amblyopia""[MeSH Terms] OR ""Amblyopia""[All Fields]) OR (""Anisometropic""[All Fields] AND ""Amblyopia""[All Fields])) OR ""anisometropic amblyopia""[All Fields])) OR (((""Amblyopia""[MeSH Terms] OR ""Amblyopia""[All Fields]) OR (""Amblyopia""[All Fields] AND ""Anisometropic""[All Fields])) OR ""amblyopia anisometropic""[All Fields])) OR (((""Amblyopia""[MeSH Terms] OR ""Amblyopia""[All Fields]) OR (""Amblyopias""[All Fields] AND ""Anisometropic""[All Fields])) OR ""amblyopias anisometropic""[All Fields])) OR (((""Amblyopia""[MeSH Terms] OR ""Amblyopia""[All Fields]) OR (""Anisometropic""[All Fields] AND ""Amblyopias""[All Fields])) OR ""anisometropic amblyopias""[All Fields])) OR (((""Amblyopia""[MeSH Terms] OR ""Amblyopia""[All Fields]) OR (""Amblyopia""[All Fields] AND ""Developmental""[All Fields])))) OR (((""Amblyopia""[MeSH Terms] OR ""Amblyopia""[All Fields]) OR (""Amblyopias""[All Fields] AND ""Developmental""[All Fields])))) OR (((""Amblyopia""[MeSH Terms] OR ""Amblyopia""[All Fields]) OR (""Developmental""[All Fields] AND ""Amblyopia""[All Fields])))) OR (((""Amblyopia""[MeSH Terms] OR ""Amblyopia""[All Fields]) OR (""Developmental""[All Fields] AND ""Amblyopias""[All Fields])))) OR (((""Amblyopia""[MeSH Terms] OR ""Amblyopia""[All Fields]) OR (""Amblyopia""[All Fields]

AND ""Suppression""[All Fields])) OR ""amblyopia suppression""[All Fields])) OR (((""Amblyopia""[MeSH Terms] OR ""Amblyopia""[All Fields]) OR (""Amblyopias""[All Fields] AND ""Suppression""[All Fields])))) OR (((""Amblyopia""[MeSH Terms] OR ""Amblyopia""[All Fields]) OR (""Suppression""[All Fields] AND ""Amblyopia""[All Fields])) OR ""suppression amblyopia""[All Fields])) OR (((""Amblyopia""[MeSH Terms] OR ""Amblyopia""[All Fields]) OR (""Suppression""[All Fields] AND ""Amblyopias""[All Fields])))) OR (((""Amblyopia""[MeSH Terms] OR ""Amblyopia""[All Fields]) OR (((""Stimulus""[All Fields] AND ""Deprivation""[All Fields]) AND ""Induced""[All Fields]) AND ""Amblyopia""[All Fields])))) OR (((""Amblyopia""[MeSH Terms] OR ""Amblyopia""[All Fields]) OR (((""Stimulus""[All Fields] AND ""Deprivation""[All Fields]) AND ""Induced""[All Fields]) AND ""Amblyopia""[All Fields])))) OR (((""Amblyopia""[MeSH Terms] OR ""Amblyopia""[All Fields]) OR ""Amblyopias""[All Fields]) AND (""Stimulus""[All Fields] OR ""stimulus's""[All Fields]) AND ""Deprivation-Induced""[All Fields])) OR (((""Amblyopia""[MeSH Terms] OR ""Amblyopia""[All Fields]) OR (((""Amblyopia""[All Fields] AND ""Stimulus""[All Fields]) AND ""Deprivation""[All Fields]) AND ""Induced""[All Fields])))) OR (((""Amblyopia""[MeSH Terms] OR ""Amblyopia""[All Fields]) OR ""Amblyopias""[All Fields]) AND (""Stimulus""[All Fields] OR ""stimulus's""[All Fields]) AND ""Deprivation-Induced""[All Fields])) OR (""Deprivation-Induced""[All Fields] AND ((""Amblyopia""[MeSH Terms] OR ""Amblyopia""[All Fields]) OR ""Amblyopias""[All Fields]) AND (""Stimulus""[All Fields] OR ""stimulus's""[All Fields]))) OR (""Deprivation-Induced""[All Fields] AND ((""Amblyopia""[MeSH Terms] OR ""Amblyopia""[All Fields]) OR ""Amblyopias""[All Fields]) AND (""Stimulus""[All Fields] OR ""stimulus's""[All Fields]))) OR (((""Amblyopia""[MeSH Terms] OR ""Amblyopia""[All Fields]) OR (((""Stimulus""[All Fields] AND ""Deprivation""[All Fields]) AND ""Induced""[All Fields]) AND ""Amblyopias""[All Fields])))))) AND (((""prevalence""[MeSH Terms] OR (((((((((""Epidemiology""[MeSH Subheading] OR ""Epidemiology""[All Fields]) OR ""prevalence""[All Fields]) OR ""prevalence""[MeSH Terms]) OR ""prevalance""[All Fields]) OR ""prevalences""[All Fields]) OR ""prevalence's""[All Fields]) OR ""prevalent""[All Fields]) OR ""prevalently""[All Fields]) OR ""prevalents""[All Fields])) OR ((((""cross-sectional studies""[MeSH Terms] OR (""cross-sectional""[All Fields] AND ""studies""[All Fields])) OR ""cross-sectional studies""[All Fields]) OR (""prevalence""[All Fields] AND ""study""[All Fields])) OR ""prevalence study""[All Fields])) OR ((((((((((""Epidemiology""[MeSH Terms] OR ""Epidemiology""[MeSH Subheading]) OR ((((((((""epidemic's""[All Fields] OR ""epidemical""[All Fields]) OR ""epidemically""[All Fields]) OR ""epidemicity""[All Fields]) OR ""epidemics""[MeSH Terms]) OR ""epidemics""[All Fields]) OR ""epidemic""[All Fields]) OR ""Epidemiology""[MeSH Subheading]) OR ""Epidemiology""[All Fields])) OR ((((((""Epidemiology""[MeSH Subheading] OR ""Epidemiology""[All Fields]) OR ""frequency""[All Fields]) OR ""Epidemiology""[MeSH Terms]) OR ""frequence""[All Fields]) OR ""frequences""[All Fields]) OR ""frequencies""[All Fields])) OR (((((((""Epidemiology""[MeSH Subheading] OR ""Epidemiology""[All Fields]) OR ""surveillance""[All Fields]) OR ""Epidemiology""[MeSH Terms]) OR ""surveilance""[All Fields]) OR ""surveillances""[All Fields]) OR ""surveilled""[All Fields]) OR ""surveillence""[All Fields])) OR ((((((""Epidemiology""[MeSH Subheading] OR ""Epidemiology""[All Fields]) OR ""morbidity""[All Fields]) OR ""morbidity""[MeSH Terms]) OR ""morbid""[All Fields]) OR ""morbidities""[All Fields]) OR ""morbids""[All Fields])) OR ((((""Epidemiology""[MeSH Subheading] OR ""Epidemiology""[All Fields]) OR ""occurrence""[All Fields]) OR ""Epidemiology""[MeSH Terms]) OR ""occurrences""[All Fields])) OR (((((((""disease outbreaks""[MeSH Terms] OR (""disease""[All Fields] AND ""outbreaks""[All Fields])) OR

""disease outbreaks""[All Fields]) OR ""outbreak""[All Fields]) OR ""Epidemiology""[MeSH Subheading]) OR ""Epidemiology""[All Fields]) OR ""outbreaks""[All Fields]) OR ""outbreak's""[All Fields])) OR (((((((((""Epidemiology""[MeSH Subheading] OR ""Epidemiology""[All Fields]) OR ""prevalence""[All Fields]) OR ""prevalence""[MeSH Terms]) OR ""prevalance""[All Fields]) OR ""prevalences""[All Fields]) OR ""prevalence's""[All Fields]) OR ""prevalent""[All Fields]) OR ""prevalently""[All Fields]) OR ""prevalents""[All Fields])) OR ((((((((((""endemic""[All Fields] OR ""endemically""[All Fields]) OR ""endemicities""[All Fields]) OR ""endemicity""[All Fields]) OR ""endemism""[All Fields]) OR ""endemisms""[All Fields]) OR ""endemization""[All Fields]) OR ""Epidemiology""[MeSH Subheading]) OR ""Epidemiology""[All Fields]) OR ""endemics""[All Fields]) OR ""Epidemiology""[MeSH Terms])) OR ((((((""Epidemiology""[MeSH Subheading] OR ""Epidemiology""[All Fields]) OR ""incidence""[All Fields]) OR ""incidence""[MeSH Terms]) OR ""incidences""[All Fields]) OR ""incident""[All Fields]) OR ""incidents""[All Fields]))))","1,364",21:04:13
16,"((Prevalence[MeSH Terms]) OR (Prevalences) OR (prevalence study)) OR (""Epidemiology""[Mesh] OR ""epidemiology"" [Subheading] OR epidemics OR frequency OR surveillance OR morbidity OR occurrence OR outbreaks OR prevalence OR endemics OR incidence)",,,"((""prevalence""[MeSH Terms] OR (((((((((""Epidemiology""[MeSH Subheading] OR ""Epidemiology""[All Fields]) OR ""prevalence""[All Fields]) OR ""prevalence""[MeSH Terms]) OR ""prevalance""[All Fields]) OR ""prevalences""[All Fields]) OR ""prevalence's""[All Fields]) OR ""prevalent""[All Fields]) OR ""prevalently""[All Fields]) OR ""prevalents""[All Fields])) OR ((((""cross-sectional studies""[MeSH Terms] OR (""cross-sectional""[All Fields] AND ""studies""[All Fields])) OR ""cross-sectional studies""[All Fields]) OR (""prevalence""[All Fields] AND ""study""[All Fields])) OR ""prevalence study""[All Fields])) OR ((((((((((""Epidemiology""[MeSH Terms] OR ""Epidemiology""[MeSH Subheading]) OR ((((((((""epidemic's""[All Fields] OR ""epidemical""[All Fields]) OR ""epidemically""[All Fields]) OR ""epidemicity""[All Fields]) OR ""epidemics""[MeSH Terms]) OR ""epidemics""[All Fields]) OR ""epidemic""[All Fields]) OR ""Epidemiology""[MeSH Subheading]) OR ""Epidemiology""[All Fields])) OR ((((((""Epidemiology""[MeSH Subheading] OR ""Epidemiology""[All Fields]) OR ""frequency""[All Fields]) OR ""Epidemiology""[MeSH Terms]) OR ""frequence""[All Fields]) OR ""frequences""[All Fields]) OR ""frequencies""[All Fields])) OR (((((((""Epidemiology""[MeSH Subheading] OR ""Epidemiology""[All Fields]) OR ""surveillance""[All Fields]) OR ""Epidemiology""[MeSH Terms]) OR ""surveilance""[All Fields]) OR ""surveillances""[All Fields]) OR ""surveilled""[All Fields]) OR ""surveillence""[All Fields])) OR ((((((""Epidemiology""[MeSH Subheading] OR ""Epidemiology""[All Fields]) OR ""morbidity""[All Fields]) OR ""morbidity""[MeSH Terms]) OR ""morbid""[All Fields]) OR ""morbidities""[All Fields]) OR ""morbids""[All Fields])) OR ((((""Epidemiology""[MeSH Subheading] OR ""Epidemiology""[All Fields]) OR ""occurrence""[All Fields]) OR ""Epidemiology""[MeSH Terms]) OR ""occurrences""[All Fields])) OR (((((((""disease outbreaks""[MeSH Terms] OR (""disease""[All Fields] AND ""outbreaks""[All Fields])) OR ""disease outbreaks""[All Fields]) OR ""outbreak""[All Fields]) OR ""Epidemiology""[MeSH Subheading]) OR ""Epidemiology""[All Fields]) OR ""outbreaks""[All Fields]) OR ""outbreak's""[All Fields])) OR (((((((((""Epidemiology""[MeSH Subheading] OR ""Epidemiology""[All Fields]) OR ""prevalence""[All Fields]) OR ""prevalence""[MeSH Terms]) OR ""prevalance""[All Fields]) OR ""prevalences""[All Fields]) OR ""prevalence's""[All Fields]) OR ""prevalent""[All Fields]) OR ""prevalently""[All Fields]) OR

""prevalents""[All Fields])) OR ((((((((((""endemic""[All Fields] OR ""endemically""[All Fields]) OR ""endemicities""[All Fields]) OR ""endemicity""[All Fields]) OR ""endemism""[All Fields]) OR ""endemisms""[All Fields]) OR ""endemization""[All Fields]) OR ""Epidemiology""[MeSH Subheading]) OR ""Epidemiology""[All Fields]) OR ""endemics""[All Fields]) OR ""Epidemiology""[MeSH Terms])) OR ((((((""Epidemiology""[MeSH Subheading] OR ""Epidemiology""[All Fields]) OR ""incidence""[All Fields]) OR ""incidence""[MeSH Terms]) OR ""incidences""[All Fields]) OR ""incident""[All Fields]) OR ""incidents""[All Fields]))","4,736,157",21:03:01
15,"""Epidemiology""[Mesh] OR ""epidemiology"" [Subheading] OR epidemics OR frequency OR surveillance OR morbidity OR occurrence OR outbreaks OR prevalence OR endemics OR incidence",,,"(((((((((""Epidemiology""[MeSH Terms] OR ""Epidemiology""[MeSH Subheading]) OR ((((((((""epidemic's""[All Fields] OR ""epidemical""[All Fields]) OR ""epidemically""[All Fields]) OR ""epidemicity""[All Fields]) OR ""epidemics""[MeSH Terms]) OR ""epidemics""[All Fields]) OR ""epidemic""[All Fields]) OR ""Epidemiology""[MeSH Subheading]) OR ""Epidemiology""[All Fields])) OR ((((((""Epidemiology""[MeSH Subheading] OR ""Epidemiology""[All Fields]) OR ""frequency""[All Fields]) OR ""Epidemiology""[MeSH Terms]) OR ""frequence""[All Fields]) OR ""frequences""[All Fields]) OR ""frequencies""[All Fields])) OR (((((((""Epidemiology""[MeSH Subheading] OR ""Epidemiology""[All Fields]) OR ""surveillance""[All Fields]) OR ""Epidemiology""[MeSH Terms]) OR ""surveilance""[All Fields]) OR ""surveillances""[All Fields]) OR ""surveilled""[All Fields]) OR ""surveillence""[All Fields])) OR ((((((""Epidemiology""[MeSH Subheading] OR ""Epidemiology""[All Fields]) OR ""morbidity""[All Fields]) OR ""morbidity""[MeSH Terms]) OR ""morbid""[All Fields]) OR ""morbidities""[All Fields]) OR ""morbids""[All Fields])) OR ((((""Epidemiology""[MeSH Subheading] OR ""Epidemiology""[All Fields]) OR ""occurrence""[All Fields]) OR ""Epidemiology""[MeSH Terms]) OR ""occurrences""[All Fields])) OR (((((((""disease outbreaks""[MeSH Terms] OR (""disease""[All Fields] AND ""outbreaks""[All Fields])) OR ""disease outbreaks""[All Fields]) OR ""outbreak""[All Fields]) OR ""Epidemiology""[MeSH Subheading]) OR ""Epidemiology""[All Fields]) OR ""outbreaks""[All Fields]) OR ""outbreak's""[All Fields])) OR (((((((((""Epidemiology""[MeSH Subheading] OR ""Epidemiology""[All Fields]) OR ""prevalence""[All Fields]) OR ""prevalence""[MeSH Terms]) OR ""prevalance""[All Fields]) OR ""prevalences""[All Fields]) OR ""prevalence's""[All Fields]) OR ""prevalent""[All Fields]) OR ""prevalently""[All Fields]) OR ""prevalents""[All Fields])) OR ((((((((((""endemic""[All Fields] OR ""endemically""[All Fields]) OR ""endemicities""[All Fields]) OR ""endemicity""[All Fields]) OR ""endemism""[All Fields]) OR ""endemisms""[All Fields]) OR ""endemization""[All Fields]) OR ""Epidemiology""[MeSH Subheading]) OR ""Epidemiology""[All Fields]) OR ""endemics""[All Fields]) OR ""Epidemiology""[MeSH Terms])) OR ((((((""Epidemiology""[MeSH Subheading] OR ""Epidemiology""[All Fields]) OR ""incidence""[All Fields]) OR ""incidence""[MeSH Terms]) OR ""incidences""[All Fields]) OR ""incident""[All Fields]) OR ""incidents""[All Fields])","4,609,175",21:01:55

11,(Prevalence[MeSH Terms]) OR (Prevalences) OR (prevalence study),,,"(""prevalence""[MeSH Terms] OR (((((((((""epidemiology""[MeSH Subheading] OR ""epidemiology""[All Fields]) OR ""prevalence""[All Fields]) OR ""prevalence""[MeSH Terms]) OR ""prevalance""[All Fields]) OR ""prevalences""[All Fields]) OR ""prevalence's""[All Fields]) OR ""prevalent""[All Fields]) OR ""prevalently""[All Fields]) OR ""prevalents""[All Fields])) OR ((((""cross-sectional studies""[MeSH Terms] OR (""cross-sectional""[All Fields] AND ""studies""[All Fields])) OR ""cross-sectional studies""[All Fields]) OR (""prevalence""[All Fields] AND ""study""[All Fields])) OR ""prevalence study""[All Fields])","2,920,351",20:56:53
10,"(Amblyopia[MeSH Terms]) OR ((Amblyopias) OR (Lazy Eye) OR (Eye, Lazy) OR (Eyes, Lazy) OR (Lazy Eyes) OR (Anisometropic Amblyopia) OR (Amblyopia, Anisometropic) OR (Amblyopias, Anisometropic) OR (Anisometropic Amblyopias) OR (Amblyopia, Developmental) OR (Amblyopias, Developmental) OR (Developmental Amblyopia) OR (Developmental Amblyopias) OR (Amblyopia, Suppression) OR (Amblyopias, Suppression) OR (Suppression Amblyopia) OR (Suppression Amblyopias) OR (Stimulus Deprivation-Induced Amblyopia) OR (Stimulus Deprivation Induced Amblyopia) OR (Amblyopia, Stimulus Deprivation-Induced) OR (Amblyopia, Stimulus Deprivation Induced) OR (Amblyopias, Stimulus Deprivation-Induced) OR (Deprivation-Induced Amblyopia, Stimulus) OR (Deprivation-Induced Amblyopias, Stimulus) OR (Stimulus Deprivation-Induced Amblyopias))",,,"""Amblyopia""[MeSH Terms] OR (((((((((((((((((((((((((""Amblyopia""[MeSH Terms] OR ""Amblyopia""[All Fields]) OR ""Amblyopias""[All Fields]) OR (((""Amblyopia""[MeSH Terms] OR ""Amblyopia""[All Fields]) OR (""Lazy""[All Fields] AND ""Eye""[All Fields])) OR ""lazy eye""[All Fields])) OR (((""Amblyopia""[MeSH Terms] OR ""Amblyopia""[All Fields]) OR (""Eye""[All Fields] AND ""Lazy""[All Fields])) OR ""eye lazy""[All Fields])) OR (((""Amblyopia""[MeSH Terms] OR ""Amblyopia""[All Fields]) OR (""Eyes""[All Fields] AND ""Lazy""[All Fields])))) OR (((""Amblyopia""[MeSH Terms] OR ""Amblyopia""[All Fields]) OR (""Lazy""[All Fields] AND ""Eyes""[All Fields])) OR ""lazy eyes""[All Fields])) OR (((""Amblyopia""[MeSH Terms] OR ""Amblyopia""[All Fields]) OR (""Anisometropic""[All Fields] AND ""Amblyopia""[All Fields])) OR ""anisometropic amblyopia""[All Fields])) OR (((""Amblyopia""[MeSH Terms] OR ""Amblyopia""[All Fields]) OR (""Amblyopia""[All Fields] AND ""Anisometropic""[All Fields])) OR ""amblyopia anisometropic""[All Fields])) OR (((""Amblyopia""[MeSH Terms] OR ""Amblyopia""[All Fields]) OR (""Amblyopias""[All Fields] AND ""Anisometropic""[All Fields])) OR ""amblyopias anisometropic""[All Fields])) OR (((""Amblyopia""[MeSH Terms] OR ""Amblyopia""[All Fields]) OR (""Anisometropic""[All Fields] AND ""Amblyopias""[All Fields])) OR ""anisometropic amblyopias""[All Fields])) OR (((""Amblyopia""[MeSH Terms] OR ""Amblyopia""[All Fields]) OR (""Amblyopia""[All Fields] AND ""Developmental""[All Fields])))) OR (((""Amblyopia""[MeSH Terms] OR ""Amblyopia""[All Fields]) OR (""Amblyopias""[All Fields] AND ""Developmental""[All Fields])))) OR (((""Amblyopia""[MeSH Terms] OR ""Amblyopia""[All Fields]) OR (""Developmental""[All Fields] AND ""Amblyopia""[All Fields])))) OR (((""Amblyopia""[MeSH Terms] OR ""Amblyopia""[All Fields]) OR (""Developmental""[All Fields] AND ""Amblyopias""[All Fields])))) OR (((""Amblyopia""[MeSH Terms] OR ""Amblyopia""[All Fields]) OR (""Amblyopia""[All Fields] AND ""Suppression""[All Fields])) OR ""amblyopia suppression""[All Fields])) OR (((""Amblyopia""[MeSH Terms] OR ""Amblyopia""[All Fields]) OR (""Amblyopias""[All Fields] AND

""Suppression""[All Fields])))) OR (((""Amblyopia""[MeSH Terms] OR ""Amblyopia""[All Fields]) OR (""Suppression""[All Fields] AND ""Amblyopia""[All Fields])) OR ""suppression amblyopia""[All Fields])) OR (((""Amblyopia""[MeSH Terms] OR ""Amblyopia""[All Fields]) OR (""Suppression""[All Fields] AND ""Amblyopias""[All Fields])))) OR (((""Amblyopia""[MeSH Terms] OR ""Amblyopia""[All Fields]) OR (((""Stimulus""[All Fields] AND ""Deprivation""[All Fields]) AND ""Induced""[All Fields]) AND ""Amblyopia""[All Fields])))) OR (((""Amblyopia""[MeSH Terms] OR ""Amblyopia""[All Fields]) OR (((""Stimulus""[All Fields] AND ""Deprivation""[All Fields]) AND ""Induced""[All Fields]) AND ""Amblyopia""[All Fields])))) OR (((""Amblyopia""[MeSH Terms] OR ""Amblyopia""[All Fields]) OR ""Amblyopias""[All Fields]) AND (""Stimulus""[All Fields] OR ""stimulus's""[All Fields]) AND ""Deprivation-Induced""[All Fields])) OR (((""Amblyopia""[MeSH Terms] OR ""Amblyopia""[All Fields]) OR (((""Amblyopia""[All Fields] AND ""Stimulus""[All Fields]) AND ""Deprivation""[All Fields]) AND ""Induced""[All Fields])))) OR (((""Amblyopia""[MeSH Terms] OR ""Amblyopia""[All Fields]) OR ""Amblyopias""[All Fields]) AND (""Stimulus""[All Fields] OR ""stimulus's""[All Fields]) AND ""Deprivation-Induced""[All Fields])) OR (""Deprivation-Induced""[All Fields] AND ((""Amblyopia""[MeSH Terms] OR ""Amblyopia""[All Fields]) OR ""Amblyopias""[All Fields]) AND (""Stimulus""[All Fields] OR ""stimulus's""[All Fields]))) OR (""Deprivation-Induced""[All Fields] AND ((""Amblyopia""[MeSH Terms] OR ""Amblyopia""[All Fields]) OR ""Amblyopias""[All Fields]) AND (""Stimulus""[All Fields] OR ""stimulus's""[All Fields]))) OR (((""Amblyopia""[MeSH Terms] OR ""Amblyopia""[All Fields]) OR (((""Stimulus""[All Fields] AND ""Deprivation""[All Fields]) AND ""Induced""[All Fields]) AND ""Amblyopias""[All Fields]))))","9,114",20:55:22
6,((child[MeSH Terms]) OR (children)) OR (pediatric),,,"""child""[MeSH Terms] OR ""child""[MeSH Terms] OR ""child""[All Fields] OR ""children""[All Fields] OR ""child's""[All Fields] OR ""children's""[All Fields] OR ""childrens""[All Fields] OR ""childs""[All Fields] OR ""paediatrics""[All Fields] OR ""pediatrics""[MeSH Terms] OR ""pediatrics""[All Fields] OR ""paediatric""[All Fields] OR ""pediatric""[All Fields]","2,934,097",20:48:21

Supplementary Table S2. General characteristics of the included studies

Study	Country/Race	Age (year, mean or median)	Male (%)	Cause	Definition of amblyopia	No of cases	No of subjects	Prevalence (%)	95%CI
Harrington, 2019 (50)	UK	9-16	NR	Anisometropia, strabismus, mixed mechanism, and refractive error	BCVA ≥0.3 logMAR (≤6/12 Snellen, 20/40)	39	1621	2.41	1.76-3.27
Li, 2019 (51)	China	30-83months	3835 (52.13%)	Anisometropic, strabismic, deprivation, and refractive	Unilateral: BCVA ≤20/32; Bilateral: BCVA (<20/50 for 30- to 47-month-old or <20/40 for ≥48-month-old)	80	7356	1.09	0.87-1.35
Ugurbas, 2019 (52)	Turkey	7.9±1.4	NR	NR	2 line difference in the BCVA	49	2846	1.72	1.3-2.27
Zhu, 2019 (53)	China (Mojiang Hani Autonomous County)	Grade 1:7.7±0.6; Grade 7:13.8±0.7	Grade 1:904 (54.6%), Grade 7:718 (51.5%)	Strabismic, anisometropic, and ametropic	Unilateral: BCVA≤20/32 (≥0.2 logMAR); Bilatera: BCVA <20/40 (>0.3 logMAR)	25	3050	0.82	0.56-1.21
Dikova, 2018 (16)	Bulgaria	7.7 (4-10)	901 (53.8%)	Strabismic, anisometropic, isoametropic, and deprivation	Reduction of the BCVA for near and far under 40/60 on one or both eyes	42	1675	2.51	1.86-3.37
Ghaderi, 2018 (54)	Iran	7	2127 (51.8%)	NR	NR	8	4106	0.19	0.1-0.38
Hansen, 2018 (55)	Denmark	11.7±0.4	627 (47%)	Strabismus, anisometropia, and anisoastigmatism	Difference of at least 10 ETDRS letters between the two eyes and a BCVA < 80	20	1335	1.5	0.97-2.3

					letters [> 0.1 logMAR, <20/25 (0.8) Snellen] in the worse seeing eye				
Huang, 2018 (33)	China	36-48 months	900 (53.1%)	Astigmatism, SE refractive error, and SE anisometropia	Unilateral: 2 line interocular difference in BCVA, with ≤20/32 (logMAR 0.2); Bilateral: bilateral subnormal BCVA <20/50 (logMAR 0.4)	25	1695	1.47	1-2.17
Khan, 2018 (56)	Pakistan	10.2 (5,15)	609 (51.09%)	Anisometropia, meridional, strabismus, stimulus deprivation (13.9%), and combined	NR	43	1192	3.61	2.69-4.82
Magdalene, 2018 (57)	India	13.5±2	20619 (52%)	Refractive, deprivation, and strabismic	Unilateral: 2 line interocular difference in VA with a VA of at least 6/12 (fails to read 6/9 line) or worse in the worse eye (with unilateral amblyogenic factors); Bilateral: VA of 6/12 or less in both eyes (with bilateral amblyogenic factors).	692	39651	1.75	1.62-1.88
Min, 2018 (58)	Malaysia	5.03±0.77	673 (52.3%)	NR	Unilateral: an inter-ocular difference of 2 lines in best presenting VA; Bilateral: if the subject had the corresponding history of obstruction of both visual axis or significant ametropia of both eyes	97	1287	7.54	6.22-9.11
Sandfeld, 2018 (59)	Denmark	68 months (53-87)	231 (52%)	NR	≥2 lines of differences between the eyes, and/or VA worse than 0.3 logMAR (3/6 Snellen equivalent)	12	445	2.7	1.55-4.65
Azizoğlu, 2017 (8)	Turkey	6.7±2.2 (4-10)	414 (50.3%)	NR	At least two lines of uncorrectable difference in visual acuity between the two eyes and either a hyperopic difference of at least 2.00 DS in the eye with poorer acuity	15	823	1.82	1.11-2.99

					or uncorrected astigmatism of at least 1 DC in the eye (s) with poorer acuity				
Pan, 2017 (40)	China	9.55±1.93 (6-14)	4733 (51.1%)	Anisometropic, strabismic, combined strabismic/anisometropic, and refractive	Unilateral: BCVA ≤20/32 in the worse eye and a 2-line interocular difference or more in BCVA; Bilateral: bilateral subnormal BCVA<20/40	132	9263	1.43	1.2-1.69
Singh, 2017 (60)	India	5-15	2271 (47%)	NR	NR	20	4838	0.41	0.27-0.64
Uddin, 2017 (61)	Malaysia	5.47±0.64 (4-6)	473 (52.6%)	NR	Two lines of best-corrected vision between the two eyes or a corrected vision less than 6/9 or worse in the affected eye	10	900	1.11	0.6-2.03
Yekta, 2017 (62)	Iran	11.05±2.93 (6-15)	631 (54.82%)	NR	BCVA of 20/30 or worse or an interocular difference of 2 or more lines of Snellen acuity in the absence of any pathology	31	1130	2.74	1.94-3.87
Chen, 2016 (11)	China	57.89±8.573 (36–72) months	3019 (53.3%)	Anisometropic, strabismic, strabismic/anisometropic, deprivational, and refractive	Unilateral: two-line interocular difference in BCVA, with ≤20/32 in the worse eye; Bilateral: bilateral subnormal BCVA <20/40 both in children aged 36–48 months and in those 48 months and older	68	5667	1.2	0.95-1.52
Griffith, 2016 (63)	USA	6.27±0.85 (3-10.9)	0.494	NR	Visual acuity, with cycloplegic correction, was <20/40 for pre-kindergarten or ≤20/40 in older children or if there was a two-line difference between each eye	873	63841	1.37	1.28-1.46
Hameed, 2016 (64)	Pakistan	5-15	951 (57.84%)	Astigmatism, hypermetropia, and myopia	With decreased vision, which does not improve with refraction in the absence of any organic cause.	28	1644	1.7	1.18-2.45

Hendler, 2016 (65)	USA	3-5	NR	NR	Unilateral: ≥2 line interocular difference in the BCVA; bilateral: BCVA <20/50 for children <4 years old and <20/40 for children ≥4 years old	95	11260	0.84	0.69-1.03
Ikuomenisan, 2016 (66)	Nigeria	7.5±1.6 (4-16)	899 (52.8%)	Refractive, sensory deprivation, strabismic	Unilateral: ≥2-line difference in BCVA between the two eyes when the VA was <6/9 (logMAR 0.18) in the worse eye; Bilateral: BCVA in both eyes <6/12 (logMAR 0.3)	24	1702	1.41	0.95-2.09
Mehravaran, 2016 (67)	USA	4.3 (3-5)	5720 (50.8%)	NR	Unilateral: 2 or more lines difference in the BVCA; bilateral: best-corrected visual acuity was worse than 20/50 for children <4 years of age and worse than 20/40 for children ≥4 years of age	95	11076	0.86	0.7-1.05
Ojaghi, 2016 (68)	Iran	4 (2,6)	19305 (50.1%)	Refractive errors, strabismus, and other reasons	Visual acuity less than (20/30) in each eye or vision difference for more than two lines between two eyes after removing refractive defects and organic eye defects	109	75173	0.14	0.12-0.17
Pan, 2016 (69)	China	3-6	3019 (53.3%)	NR	Unilateral: 2-line interocular difference in BCVA, with ≤ 0.20 logMAR (Snellen ~ 6/9 or 20/32) in the worse eye; Bilateral: bilateral subnormal BCVA <0.30 logMAR (Snellen 6/12 or 20/40) in both eyes.	68	5776	1.18	0.93-1.49
Yekta, 2016 (70)	Iran	5.1±0.63 (4,6)	1900 (51.3%)	Anisometropic, strabismic, and isoametropic	BCVA ≤20/30, or an interocular difference of two or more lines of optotype acuity in the absence of any pathology.	15	3654	0.41	0.25-0.68
Aldebasi, 2015 (6)	Kingdom of Saudi Arabia	9.53±1.88	2573 (49.71%)	Anisometropia, high bilateral refractive	Unilateral: ≥2-line difference in best VA, when <20/30 in the worse eye and with amblyogenic factors; bilateral: BCVA in	202	5176	3.9	3.41-4.47

				error, esotropia, and exotropia	both eyes <20/40 in the presence of amblyogenic factors				
Jeong, 2015 (71)	Korea	3–6	NR	Refractive, strabismus, others	BCVA of less than 0.5 at the age of three years, BCVA of less than 0.63 at the age of four or five years in either eye or who had a difference of two or more lines between the eyes	89	35626	0.25	0.2-0.31
Maqsud, 2015 (72)	UK	4.6±5months (4-5.83 y)	NR	NR	Corrected visual acuity of 0.200 logMAR was not achieved in each eye	45	2240	2.01	1.5-2.68
Xiao, 2015 (32)	China,India,Chile,Malaysia,Nepal,South Africa	5-15	20131 (51.2%)	Anisometropic, strabismic, mixed strabismic-anisometropic, hyperopia only	BCVA of 20/40 in either eye, with tropia, anisometropia, or hyperopia	290	39321	0.74	0.66-0.83
Yamamah, 2015 (73)	Egypt	10.7±3.1 (5.5–17.0)	1047 (50.6%)	NR	Decreased vision in a structurally normal eye with the difference between BCVA 2 lines.	1	2070	0.05	0.01-0.27
Chang, 2014 (10)	China (Taiwan)	7-15	213 (52.85%)	NR	BCVA ≤0.8 without ocular diseases	4	403	0.99	0.39-2.52
Fu, 2014 (74)	China	12.4±0.6 (10-16)	0.504	Anisometropic, strabismic, others	BCVA 0.1 logMAR units (50 letters, equivalent to Snellen VA 54/5) in one or both eyes in otherwise normal eyes, or with ocular pathology that involved the eye/visual pathways, with a reduction in VA not attributable only to the ocular pathology.	52	2260	2.3	1.76-3
Fu, 2014 (34)	China	7.1±0.4	0.578	NR	Unilateral: 2-line inter-ocular difference between eyes with BCVA ≤ 20/32	27	2893	0.93	0.64-1.35

					(>logMar 0.2) in the worse eye; Bilateral: BCVA in both eyes <20/40 (>logMar 0.3), in the presence of amblyogenic factors				
Hashemi, 2014 (75)	Iran	7	NR	NR	BCVA 20/30 or less or a 2-line interocular optotype acuity difference with no pathology	63	3547	1.78	1.39-2.27
Paudel, 2014 (76)	Vietnam	12-15	NR	NR	NR	6	2238	0.27	0.12-0.58
Caca, 2013 (9)	Turkey	10.56±3.59	9944 (47.7%)	NR	BCVA of 20/40 or worse in at least one eye or at least two lines of interocular difference on Snellen chart without an apparent organic cause	554	21062	2.63	2.42-2.86
Chia, 2013 (13)	Singapore	30-72mos	850 (52%)	NR	Unilateral: the 2-line difference between eyes with VA520/30 in the worse eye, and with coexisting anisometropia, strabismus or past/present visual axis obstruction; Bilateral: VA in both eyes 520/40 (in children 48–72 months) or 520/50 (30–47.9 months), with coexisting hyperopia 4.00 D, myopia 6.00 D and stigmatism 2.50 D, or past/present visual axis obstruction.	20	1682	1.19	0.77-1.83
de Koning, 2013 (15)	Netherlands	7	NR	NR	VA >0.3 logMAR	100	2964	3.37	2.78-4.09
Ganekal, 2013 (77)	USA	5-15	2105 (52%)	NR	BCVA 6/12 in the affected eye without any underlying structural abnormality of the visual pathway, a 2-line difference	44	4020	1.09	0.82-1.47

					between the two eyes, and the presence of an amblyogenic factor				
Gursoy, 2013 (78)	Turkey	7-8	362 (51.06%)	Anisometropic, isometropic, strabismic	The difference in SEq between the two eyes of 41.00 D.	39	709	5.5	4.05-7.43
McKean-Cowdin, 2013 (41)	USA	30-72mos	969 (51.46%)	Anisometropic, strabismic, anisometropic, and strabismic, deprivational	Unilateral: 2-line interocular difference in best-corrected VA with ≤20/32 in the worse eye; Bilateral: bilaterally decreased best-corrected VA (<20/50 for ≥30- to 47-month-olds or <20/40 for ≥48-month-olds) or with evidence of visual axis obstruction of both eyes	34	1875	1.81	1.3-2.52
Moraes Ibrahim, 2013 (79)	Brazil	10-15	814 (51%)	NR	NR	4	1590	0.25	0.1-0.65
Sapkota, 2013 (80)	USA	7.74±2.97 (3-13)	NR	Strabismic, refractive, stimulus deprivation	BCVA was 20/30 or worse; mild (with BCVA of 20/30 to 20/40), moderate (with BCVA 20/60 – 20/80), or severe (with BCVA of less than 20/80)	440	62633	0.7	0.64-0.77
Wu, 2013 (81)	China	9.7±3.3 (4-18)	3186 (52.9%)	NR	BCVA ≤20/32	44	6026	0.73	0.54-0.98
Pai, 2012 (82)	Australian	30-72months	745 (52.39%)	NR	Multi-Ethnic Pediatric Eye Disease Study (MEPEDS) criteria	27	1422	1.9	1.31-2.75
Pi, 2012 (83)	China	10.41±2.73	1615 (52.45%)	Ametropic, anisometropic, strabismic, perception	BCVA in 1 or both eyes was lower than the normal visual acuity for children of the same age in the absence of organic changes.	58	3079	1.88	1.46-2.43

Polling, 2012 (84)	Netherlands	7 (2mon-12y)	301 (51%)	Refractive amblyopia, strabismic amblyopia, a combination of strabismus and anisometropia	BCVA >=0.3 (<=20/40) LogMAR in the affected eye, together with a 2 LogMAR line difference between the two eyes and the presence of an amblyogenic factor.	13	420	3.1	1.82-5.22
Faghihi, 2011 (20)	Iran	13.2 ± 3.2 (6-21)	1208 (56.2%)	NR	BCVA of 20/30 or worse, or a 2-line interocular optotype acuity difference in the absence of any pathology.	41	2150	1.91	1.41-2.58
Sherpa, 2011 (85)	Nepal	0-15	223 (47.85%)	NR	vision was 6/9 or worse	2	466	0.43	0.12-1.55
Al-Rowaily, 2010 (7)	Saudi Arabia	4-6	577 (43.7%)	NR	Children with a visual acuity of 20/28 or worse in one or both eyes	6	1319	0.45	0.21-0.99
Chia, 2010 (12)	Singapore	30-72months	850 (50.5%)		Unilateral:2-line difference in best VA, when20/30 (logMAR 0.18) in the worse eye	20	1682	1.19	0.77-1.83
Marasini, 2010 (86)	Nepal	10.78±3.61 (3-12)	843 (46.8%)	NR	could not be corrected to 20/30 or better	6	1802	0.33	0.15-0.72
Yekta, 2010 (46)	Iran	12.50±3.00 (7-17)	1341 (49.98%)	NR	BCVA 20/30 or less or a 2-line interocular optotype acuity difference with no pathology	62	2683	2.31	1.81-2.95
Friedman, 2009 (87)	USA	30-71months	NR	NR	Unilateral:2-line interocular difference (IOD) in BCVA, 20/32 or worse in the worse ey; Bilateral: bilateral subnormal BCVA (worse than 20/50 in children aged 30 to 47 months, or worse than 20/40 in	19	1546	1.23	0.79-1.91

					children ≥48 months) hyperopia, ≥6.00 D SE myopia, or ≥2.50 D astigmatism).				
Huynh, 2009 (88)	Australia	6.7± 0.4--12.7.7± 0.4years	1815 (51.4%)	NR	NR	65	3529	1.84	1.45-2.34
Jamali, 2009 (47)	Iran	6	436 (53.5%)	NR	BCVA equal or 6/12 in either eye or a difference of two or more lines of BCVA between the two eyes without an organic cause.	14	815	1.72	1.03-2.86
Lai, 2009 (89)	China (Taiwan)	3-6	314 (50.8%)	NR	BCVA is 2 or more lines on the chart, or BCVA is worse than 0.6 at age 4 years, 0.7 at age 5 years, or 0.8 at age 6 years.		618	6.31	4.65-8.51
Unsal, 2009 (90)	Turkey	10.52±2.28 (6-17)	864 (53.7%)	NR	NR	81	1606	5.04	4.08-6.23
Drover, 2008 (18)	Canada	4.2±1.1	NR	NR	Unilateral >1 line difference in acuity; Bilatera: 2- and 3-year-olds <20/50 in 1 eye, <20/40 in the contralateral eye, 4-year-olds and older <20/40 in 1 eye, <20/30 in the contralateral eye	44	946	4.65	3.48-6.19
Lu, 2008 (91)	China (Tibetan)	10.50±1.91	645 (59.5%)	NR	6/9 or worse	11	1084	1.01	0.57-1.81
Multi-ethnic Pediatric Eye Disease Study Group, 2008 (38)	USA	30-72months	NR	NR	Unilateral: 2-line interocular difference in BCVA with 20/32 or worse in the worse eye	69	3350	2.06	1.63-2.6

Robaei, 2008 (92)	Australia	12.7 (11.1-14.4)	1190 (50.57%)	NR		Various criteria of BCVA together with an amblyogenic factor and absence of significant organic pathology.	44	2353	1.87	1.4-2.5
Salomão, 2008 (93)	Brazil	11-14	1137 (46.6%)	NR		Unilateral visual impairment (20/32 or better in one eye only), mild impairment in the better eye (20/40–20/63 in the better eye), moderate impairment in the better eye (20/80–20/160 in the better eye),	25	2438	1.03	0.7-1.51
Sapkota, 2008 (94)	Nepal	10-15	2278 (53.2%)	NR		NR	17	4282	0.4	0.25-0.63
Williams, 2008 (35)	England	7	3858 (50.8%)	NR		History of patching treatment and/or with an interocular difference in best acuity for each eye of > 0.2 LogMAR units where the worst-seeing eye had the best acuity of worse than 0.3 LogMAR and the eye looked normal on dilated fundoscopy	272	7538	3.61	3.21-4.05
He, 2007 (95)	China	13-17	NR	NR		BCVA ≤0/40 and no apparent organic lesion	12	2400	0.5	0.29-0.87
Matsuo, 2007 (96)	Japan	3 (3.5-4)	NR	NR		NR	53	33193	0.16	0.12-0.21
Matsuo, 2007 (97)	Japan	6-11	NR	NR		NR	173	84619	0.2	0.18-0.24
Robaei, 2006 (98)	Australian	6.7 (5.5-8.4)	880 (50.6%)	NR		Corrected VA less than 0.3 logMAR unit (i.e., <40 letters, equivalent to Snellen VA <20/40) in the affected eye not attributable to any underlying structural abnormality of the eye or visual pathway plus a difference	32	1739	1.84	1.31-2.59

					of at least 2 logMAR lines between the 2 eyes.				
Donnelly, 2005 (17)	UK	4.3±1.94	NR	NR	NR	18	1582	1.14	0.72-1.79
Matsuo, 2005 (99)	Japan	6-11	NR	NR	The diagnostic entities in amblyopia were based on the textbook classification: anisometropic, ametropic, strabismic, and form deprivation amblyopia.	125	86531	0.14	0.12-0.17
The Vision in Preschoolers Study Group, 2005 (100)	USA	3-5	NR	NR	Unilateral: 3-line (presumed amblyopia) or 2-line (suspected amblyopia) interocular acuity difference accompanied by strabismus and/or anisometropia; bilateral: reduced VA and an amblyogenic factor in each eye	77	1452	5.3	4.26-6.58
He, 2004 (101)	China	5-15	NR	NR	NR	38	4364	0.87	0.64-1.19
Lim, 2004 (102)	South Korea	3-5	NR	NR	NR	149	35226	0.42	0.36-0.5
Tananuvat, 2004 (103)	Thailand	6-7	NR	NR	The difference in visual acuity of one line or more between eyes with the best correction and no appearance of an organic lesion.	87	6898	1.26	1.02-1.55
The Vision in Preschoolers Study Group, 2004 (104)	USA	3-5	1297 (50.1%)	NR	Presumed unilateral: 3-line interocular difference in VA and a unilateral amblyogenic factor; suspected unilateral: 2-line interocular difference in VA and a unilateral amblyogenic factor; suspected bilateral: a. 3-year-olds: worse than 20/50	163	2588	6.3	5.43-7.3

					in one eye, worse than 20/40 in the contralateral eye, and a bilateral amblyogenic factor; b. 4- and 5-year-olds: worse than 20/40 in one eye, worse than 20/30 in the contralateral eye, and a bilateral amblyogenic factor				
Maaita, 2003 (105)	Jordan	6-14	NR	NR	NR	5	1725	0.29	0.12-0.68
Williams, 2003 (106)	UK	7.5	2915 (47.9%)	NR	A: 0.2+ logMAR or more between best acuity of each eye B: the worse eye sees worse than 0.3 logMAR (worse than 6/12 on a Snellen chart)<br>C: worse eye sees 0.18 logMAR or worse (6/9 or worse on a Snellen chart)	373	6081	6.13	5.56-6.77
Dandona, 2002 (14)	India	7-15	2113 (51.87%)	NR	BCVA of 20/40 or worse and no apparent organic lesion	31	4074	0.76	0.54-1.08
Murthy, 2002 (107)	India	5-15	NR	NR	BCVA of 20/40 or worse and no apparent organic lesion	34	6447	0.53	0.38-0.74
Ohlsson, 2001 (39)	Sweden	12-13	533 (51.0%)	NR	VA of <1.0 in one eye, at least two lines of interocular difference, and no apparent organic cause.	29	1046	2.77	1.94-3.95
Eibschitz-Tsimhoni, 2000 (19)	Israel	8	NR	NR	Corrected visual acuity of ≤5/10 (20/40), or >1 line difference in corrected visual acuity between the two eyes.	41	1590	2.58	1.91-3.48
Lim, 2000 (108)	Singapore	4-4.5	NR	NR	6/12 or worse for the best-corrected vision, or at least 2 Snellen line differences in the visual acuity between the 2 eyes	8	450	1.78	0.9-3.47

Newman, 2000 (109)	UK	5.5	NR	NR	NR	17	754	2.25	1.41-3.58
Thorburn, 2000 (110)	England	5	NR	NR	NR	33	2423	1.36	0.97-1.91
Wedner, 2000 (111)	Tanzania	7-19	NR	NR	NR	3	1386	0.22	0.07-0.63
Zhao, 2000 (112)	Chile, China, and Nepal	5-15	3007 (51.1%)	NR	NR	54	5884	0.92	0.7-1.2
Lithander, 1998 (44)	Oman	6 and 12 (Grades 1 and 6)	3196 (50.8%)	NR	NR	58	6292	0.92	0.71-1.19
Kalikivayi, 1997 (113)	India	9.3±3.4 (3-18)	2121 (58.0)	NR	NR	40	3559	1.12	0.83-1.53
Martínez, 1997 (114)	Spain	3-6	667 (56.6%)	NR	The difference in visual acuity of 0.2 or more between eyes	88	1179	7.46	6.1-9.11
Preslan, 1996 (115)	USA	4-7	351 (51.6%)	NR	NR	27	680	3.97	2.74-5.72
Rosenberg, 1996 (116)	Nordic countries (Denmark, Iceland, Finland, Norway,Southern Sweden)	0-17	NR	NR	ICD-9	70	3818001	0	0-0

Abolfotouh, 1994 (5)	Saudi Arabia	13-21	3521 (100%)	NR	NR	58	3590	1.62	1.25-2.08
Jensen, 1986 (117)	Denmark	5-13	4529 (51.6%)	NR	NR	94	8769	1.07	0.88-1.31
Yassur, 1972 (118)	Rwanda	10-18	1130 (72.9%)	NR	NR	18	1550	1.16	0.74-1.83
Flom, 1966 (119)	USA	grades 1 through 6, kindergarteners	NR	NR	Monocular visual acuity of 20/40 or worse when the refractive error was neutralized by lenses, and there was a difference in acuity between the two eyes of more than one Snellen line	29	2733	1.06	0.74-1.52

BCVA: best-corrected visual acuity; UK: United Kingdom; USA: United States of America; NR: not reported.

Supplemental Table S3a. Quality assessment of included studies based on AHRQ

Study	Define the source of informatio n (survey, record review)	List inclusion and exclusion criteria for exposed and unexposed subjects (cases and controls) or refer to previous publications	Indicate period used for identifyin g patients	Indicate whether or not subjects were consecutive if not population-based	Indicate if evaluators of subjective components of the study were masked to other aspects of the status of the participants	Describe any assessments undertaken for quality assurance purposes (e.g., test/retest of primary outcome measurements)	Explain any patient exclusion s from the analysis	Describe how confoundin g was assessed and/or controlled	If applicable, explain how missing data were handled in the analysis	Summarize patient response rates and completen ess of data collection	Clarify what follow-up, if any, was expected and the percentage of patients for which incomplete data or follow-up was obtained
Harrington, 2019 (49)	Yes	Yes	Yes	Yes	Unclear	Unclear	Yes	Yes	Yes	Yes	Yes
Li, 2019 (50)	Yes	Yes	No	Yes	Unclear	Unclear	Yes	Yes	Yes	Yes	Yes
Ugurbas, 2019 (51)	Yes	Yes	Yes	Yes	Unclear	Unclear	Unclear	Yes	Yes	Unclear	Yes
Dikova, 2018 (16)	Yes	Yes	Yes	Yes	Unclear	Unclear	No	Yes	Yes	No	Yes
Ghaderi, 2018 (53)	Yes	Yes	Yes	Yes	Unclear	Unclear	Yes	Yes	Yes	Yes	Yes
Huang, 2018 (32)	Yes	Yes	Yes	Yes	Unclear	Unclear	Yes	Yes	Yes	Yes	Yes
Khan, 2018 (55)	Yes	Yes	Yes	No	Unclear	Unclear	No	Yes	Yes	No	Yes
Magdalene, 2018 (56)	Yes	Yes	Yes	No	Unclear	Unclear	No	Yes	Yes	Yes	Yes
Min, 2018 (57)	Yes	Yes	Yes	Yes	Unclear	Unclear	Yes	Yes	Yes	Yes	Yes

Sandfeld, 2018 (58)	Yes	Yes	Yes	Yes	Unclear	Unclear	Yes	Yes	Yes	Yes	Yes
Azizoğlu, 2017 (8)	Yes	Yes	Yes	Yes	Unclear	Unclear	Yes	Yes	Yes	Yes	Yes
Pan, 2017 (39)	Yes	Yes	Yes	Yes	Unclear	Unclear	Yes	Yes	Yes	Yes	Yes
Singh, 2017 (59)	Yes	Yes	Yes	Yes	Unclear	Unclear	No	Yes	Yes	No	Yes
Uddin, 2017 (60)	Yes	Yes	Yes	Yes	Unclear	Unclear	No	Yes	Yes	No	Yes
Yekta, 2017 (61)	Yes	Yes	Yes	Yes	Unclear	Unclear	No	Yes	Yes	Yes	Yes
Chen, 2016 (11)	Yes	Yes	Yes	Yes	Unclear	Unclear	Yes	Yes	Yes	Yes	Yes
Griffith, 2016 (62)	Yes	Yes	Yes	Yes	Unclear	Unclear	Yes	Yes	Yes	Yes	Yes
Hameed, 2016 (63)	Yes	Yes	Yes	Unclear	Unclear	Unclear	Yes	Yes	Yes	Yes	Yes
Hendler, 2016 (64)	Yes	Yes	Yes	Unclear	Unclear	Unclear	Yes	Yes	Yes	Yes	Yes
Ikuomenisan, 2016 (65)	Yes	Yes	Yes	Yes	Unclear	Unclear	Yes	Yes	Yes	Yes	Yes
Mehravaran, 2016 (66)	Yes	Yes	Yes	Yes	Unclear	Unclear	Unclear	Yes	Yes	Yes	Yes
Ojaghi, 2016 (67)	Yes	Yes	Yes	Yes	Unclear	Unclear	Yes	Yes	Yes	Yes	Yes
Pan, 2016 (68)	Yes	Yes	Yes	Yes	Unclear	Unclear	Yes	Yes	Yes	Yes	Yes
Yekta, 2016 (69)	Yes	Yes	Yes	Yes	Unclear	Unclear	Yes	Yes	Yes	Yes	Yes

Aldebasi, 2015 (6)	Yes	Yes	Yes	Yes	Unclear	Unclear	Yes	Yes	Yes	No	Yes
Jeong, 2015 (70)	Yes	Yes	Yes	Yes	Unclear	Unclear	Unclear	Yes	Yes	Yes	Yes
Maqsud, 2015 (71)	Yes	Yes	Yes	Unclear	Unclear	Unclear	Yes	Yes	Yes	Yes	Yes
Xiao, 2015 (31)	Yes	Yes	Unclear	Yes	Unclear	Unclear	Unclear	Yes	Yes	Yes	Yes
Yamamah, 2015 (72)	Yes	Yes	Yes	Yes	Unclear	Unclear	No	Yes	Yes	No	Yes
Chang, 2014 (10)	Yes	Yes	Yes	Yes	Unclear	Unclear	Yes	Yes	Yes	No	Yes
Fu, 2014 (33)	Yes	Yes	Yes	Yes	Unclear	Unclear	Yes	Yes	Yes	Yes	Yes
Fu, 2014 (73)	Yes	Yes	Yes	Yes	Unclear	Unclear	Yes	Yes	Yes	Yes	Yes
Hashemi, 2014 (74)	Yes	Yes	Yes	Yes	Unclear	Unclear	Yes	Yes	Yes	Yes	Yes
Paudel, 2014 (75)	Yes	Yes	Yes	Yes	Unclear	Unclear	Unclear	Yes	Yes	Yes	Yes
Caca, 2013 (9)	Yes	Yes	Yes	Yes	Unclear	Unclear	Yes	Yes	Yes	Yes	Yes
Chia, 2013 (13)	Yes	Yes	Yes	Yes	Unclear	Unclear	Unclear	Yes	Yes	Yes	Yes
Ganekal, 2013 (76)	Yes	Yes	Yes	Yes	Unclear	Unclear	Yes	Yes	Yes	No	Yes
Gursoy, 2013 (77)	Yes	Yes	Yes	Yes	Unclear	Unclear	Yes	Yes	Yes	Yes	Yes

McKean-Cowdin, 2013 (40)	Yes	Yes	Yes	Yes	Unclear	Unclear	Yes	Yes	Yes	Yes	Yes
Moraes Ibrahim, 2013 (78)	Yes	Yes	Yes	Yes	Unclear	Unclear	Yes	Yes	Yes	Yes	Yes
Sapkota, 2013 (79)	Yes	Yes	Yes	Unclear	Unclear	Unclear	Yes	Yes	Yes	Unclear	Yes
Wu, 2013 (80)	Yes	Yes	Yes	Yes	Unclear	Unclear	Yes	Yes	Yes	Yes	Yes
Pai, 2012 (81)	Yes	Yes	Yes	Yes	Unclear	Unclear	Yes	Yes	Yes	Yes	Yes
Pi, 2012 (82)	Yes	Yes	Yes	Yes	Unclear	Unclear	Yes	Yes	Yes	Yes	Yes
Polling, 2012 (83)	Yes	Yes	No	Unclear	Unclear	Unclear	Unclear	Yes	Yes	Yes	Yes
Faghihi, 2011 (20)	Yes	Yes	Yes	No	Unclear	Unclear	Yes	Yes	Yes	Yes	Yes
Sherpa, 2011 (84)	Yes	Yes	No	Yes	Unclear	Unclear	Yes	Yes	Yes	No	Yes
Al-Rowaily, 2010 (7)	Yes	Yes	Yes	Yes	Unclear	Unclear	Yes	Yes	Yes	Yes	Yes
Chia, 2010 (12)	Yes	Yes	No	Yes	Unclear	Unclear	Yes	Yes	Yes	Yes	Yes
Marasini, 2010 (85)	Yes	Yes	Yes	No	Unclear	Unclear	Yes	Yes	Yes	Unclear	Yes
Yekta, 2010 (45)	Yes	Yes	Yes	Yes	Unclear	Unclear	Unclear	Yes	Yes	Yes	Yes
Friedman, 2009 (86)	Yes	Yes	Unclear	Yes	Unclear	Unclear	Yes	Yes	Yes	Yes	Yes

Huynh, 2009 (87)	Yes	Yes	Yes	Yes	Unclear	Unclear	Unclear	Yes	Yes	Yes	Yes
Jamali, 2009 (46)	Yes	Yes	Yes	Yes	Unclear	Unclear	Yes	Yes	Yes	Yes	Yes
Lai, 2009 (88)	Yes	Yes	Yes	Yes	Unclear	Unclear	Yes	Yes	Yes	Yes	Yes
Unsal, 2009 (89)	Yes	Yes	Yes	Yes	Unclear	Unclear	Yes	Yes	Yes	Yes	Yes
Drover, 2008 (18)	Yes	Yes	Yes	Yes	Unclear	Unclear	Unclear	Yes	Yes	Yes	Yes
Lu, 2008 (90)	Yes	Yes	Yes	Yes	Unclear	Unclear	Yes	Yes	Yes	Yes	Yes
Multi-ethnic Pediatric Eye Disease Study, Group, 2008 (37)	Yes	Yes	Unclear	Yes	Unclear	Unclear	Yes	Yes	Yes	Yes	Yes
Robaei, 2008 (91)	Yes	Yes	Yes	Yes	Unclear	Unclear	Yes	Yes	Yes	Yes	Yes
Salomão, 2008 (92)	Yes	Yes	Yes	Yes	Unclear	Unclear	Unclear	Yes	Yes	Yes	Yes
Sapkota, 2008 (93)	Yes	Yes	Yes	Yes	Unclear	Unclear	Unclear	Yes	Yes	Yes	Yes
Williams, 2008 (34)	Yes	Yes	Yes	Yes	Unclear	Unclear	Yes	Yes	Yes	Yes	Yes
He, 2007 (94)	Yes	Yes	Yes	Yes	Unclear	Unclear	Yes	Yes	Yes	Yes	Yes
Matsuo, 2007 (95)	Yes	Yes	Yes	Yes	Unclear	Unclear	Unclear	Yes	Yes	Yes	Yes
Matsuo, 2007 (96)	Yes	Yes	Yes	Yes	Unclear	Unclear	Yes	Yes	Yes	Yes	Yes

Robaei, 2006 (97)	Yes	Yes	Yes	Yes	Unclear	Unclear	Yes	Yes	Yes	Yes	Yes
Donnelly, 2005 (17)	Yes	Yes	Yes	Yes	Unclear	Unclear	Yes	Yes	Yes	Unclear	Yes
Matsuo, 2005 (98)	Yes	Yes	Yes	Yes	Unclear	Unclear	Yes	Yes	Yes	Yes	Yes
The Vision in Preschoolers Study Group, 2005 (99)	Yes	Yes	Yes	Yes	Unclear	Unclear	Yes	Yes	Yes	Yes	Yes
He, 2004 (100)	Yes	Yes	Yes	Yes	Unclear	Unclear	Yes	Yes	Yes	Yes	Yes
Lim, 2004 (101)	Yes	Yes	Yes	Yes	Unclear	Unclear	Yes	Yes	Yes	Yes	Yes
Tananuvat, 2004 (102)	Yes	Yes	Yes	Unclear	Unclear	Unclear	Unclear	Yes	Yes	No	Yes
The Vision in Preschoolers Study Group, 2004 (103)	Yes	Yes	Yes	Yes	Unclear	Unclear	Yes	Yes	Yes	Yes	Yes
Maaita, 2003 (104)	Yes	Yes	Yes	Unclear	Unclear	Unclear	No	Yes	Yes	No	Yes
Williams, 2003 (105)	Yes	Yes	Yes	Yes	Unclear	Unclear	Yes	Yes	Yes	Yes	Yes
Dandona, 2002 (14)	Yes	Yes	Yes	Yes	Unclear	Unclear	Yes	Yes	Yes	Yes	Yes
Murthy, 2002 (106)	Yes	Yes	Yes	Yes	Unclear	Unclear	Yes	Yes	Yes	Yes	Yes
Ohlsson, 2001 (38)	Yes	Yes	Yes	Yes	Unclear	Unclear	Yes	Yes	Yes	No	Yes

Eibschitz-Tsimhoni, 2000 (19)	Yes	Yes	Yes	Yes	Unclear	Unclear	Yes	Yes	Yes	Yes	Yes
Lim, 2000 (107)	Yes	Yes	Yes	Yes	Unclear	Unclear	Yes	Yes	Yes	Unclear	Yes
Thorburn, 2000 (109)	Yes	Yes	Yes	Unclear	Unclear	Unclear	Yes	Yes	Yes	Yes	Yes
Wedner, 2000 (110)	Yes	Yes	Yes	Yes	Unclear	Unclear	Unclear	Yes	Yes	Yes	Yes
Zhao, 2000 (111)	Yes	Yes	Yes	Yes	Unclear	Unclear	Yes	Yes	Yes	Yes	Yes
Lithander, 1998 (43)	Yes	Yes	Yes	Yes	Unclear	Unclear	Yes	Yes	Yes	Yes	Yes
Kalikivayi, 1997 (112)	Yes	Yes	Yes	Yes	Unclear	Unclear	No	Yes	Yes	Yes	Yes
Martínez, 1997 (113)	Yes	Yes	Unclear	Yes	Unclear	Unclear	Yes	Yes	Yes	Yes	Yes
Preslan, 1996 (114)	Yes	Yes	Yes	Unclear	Unclear	Unclear	Yes	Yes	Yes	Yes	Yes
Rosenberg, 1996 (115)	Yes	Yes	Yes	Yes	Unclear	Unclear	Yes	Yes	Yes	Yes	Yes
Abolfotouh, 1994 (5)	Yes	Yes	Yes	Yes	Unclear	Unclear	No	Yes	Yes	No	Yes
Jensen, 1986 (116)	Yes	Yes	Yes	Yes	Unclear	Unclear	Unclear	Yes	Yes	No	Yes
Yassur, 1972 (117)	Yes	Yes	No	Yes	Unclear	Unclear	Yes	Yes	Yes	No	Yes
Flom, 1966 (118)	Yes	Yes	Yes	Yes	Unclear	Unclear	Yes	Yes	Yes	Yes	Yes

The quality assessment of the cross-sectional studies was performed using the Healthcare Research and Quality (AHRQ) tool, and the cohort studies were evaluated according to the Newcastle-Ottawa scale (NOS).

Supplemental Table S3b. Quality assessment of included studies based on NOS

Study	Representativeness of the exposed cohort	Selection of the non exposed cohort	Ascertainment of exposure	Demonstration that outcome of interest was not present at the start of the study	Comparability of cohorts based on the design or analysis	Assessment of outcome	Was follow-up long enough for outcomes to occur	Adequacy of follow up of cohorts	Total quality scores
Zhu, 2019 (52)	☆	☆	☆	☆	☆☆	☆	☆	☆	9
Hansen, 2018 (54)	☆	☆	☆	☆	☆☆	☆	☆	☆	9
de Koning, 2013 (15)	☆	☆	☆	☆	☆☆	☆	☆	☆	9
Newman, 2000 (108)	☆	☆	☆	☆	☆	☆	☆	☆	8

The quality assessment of the cross-sectional studies was performed using the Healthcare Research and Quality (AHRQ) tool, and the cohort studies were evaluated according to the Newcastle-Ottawa scale (NOS).

Supplemental Figure S1. Forest plot of prevalence of amblyopia of Asia (A), Africa (B), Europe (C), North America (D), Oceania (E), South America (F), and mixed countries (G). "Mixed country" referred to studies that included countries from different continents.

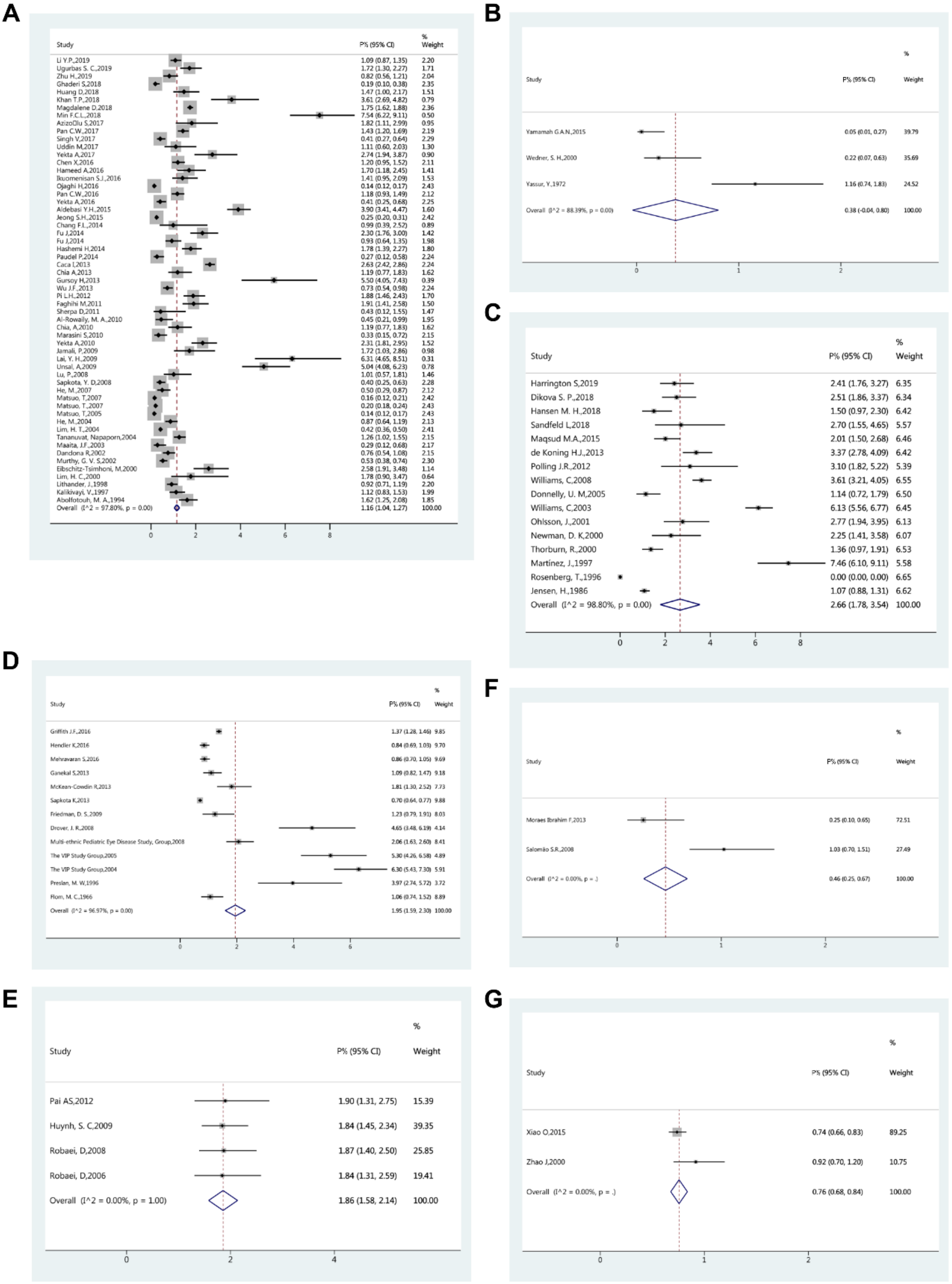

Supplement: Supplementary file 1 [file Data_Sheet_1.pdf]
